# Treatment strategies for intrauterine adhesion: focus on the exosomes and hydrogels

**DOI:** 10.3389/fbioe.2023.1264006

**Published:** 2023-08-31

**Authors:** Fengling Wu, Ningjing Lei, Shenyu Yang, Junying Zhou, Mengyu Chen, Cheng Chen, Luojie Qiu, Ruixia Guo, Yong Li, Lei Chang

**Affiliations:** ^1^ Department of Obstetrics and Gynecology, The First Affiliated Hospital of Zhengzhou University, Zhengzhou, Henan, China; ^2^ School of Basic Medical Sciences, Zhengzhou University, Zhengzhou, Henan, China; ^3^ Medical 3D Printing Center, The First Affiliated Hospital of Zhengzhou University, Zhengzhou, Henan, China; ^4^ Department of Gynaecology and Obstetrics, Chongqing General Hospital, Chongqing, China; ^5^ St George and Sutherland Clinical Campuses, School of Clinical Medicine, UNSW Sydney, Kensington, NSW, Australia

**Keywords:** intrauterine adhesion, treatment, exosome, hydrogel, 3D printing

## Abstract

Intrauterine adhesion (IUA), also referred to as Asherman Syndrome (AS), results from uterine trauma in both pregnant and nonpregnant women. The IUA damages the endometrial bottom layer, causing partial or complete occlusion of the uterine cavity. This leads to irregular menstruation, infertility, or repeated abortions. Transcervical adhesion electroreception (TCRA) is frequently used to treat IUA, which greatly lowers the prevalence of adhesions and increases pregnancy rates. Although surgery aims to disentangle the adhesive tissue, it can exacerbate the development of IUA when the degree of adhesion is severer. Therefore, it is critical to develop innovative therapeutic approaches for the prevention of IUA. Endometrial fibrosis is the essence of IUA, and studies have found that the use of different types of mesenchymal stem cells (MSCs) can reduce the risk of endometrial fibrosis and increase the possibility of pregnancy. Recent research has suggested that exosomes derived from MSCs can overcome the limitations of MSCs, such as immunogenicity and tumorigenicity risks, thereby providing new directions for IUA treatment. Moreover, the hydrogel drug delivery system can significantly ameliorate the recurrence rate of adhesions and the intrauterine pregnancy rate of patients, and its potential mechanism in the treatment of IUA has also been studied. It has been shown that the combination of two or more therapeutic schemes has broader application prospects; therefore, this article reviews the pathophysiology of IUA and current treatment strategies, focusing on exosomes combined with hydrogels in the treatment of IUA. Although the use of exosomes and hydrogels has certain challenges in treating IUA, they still provide new promising directions in this field.

## 1 Introduction

IUA causes excessive damage to the endometrium, which results in the formation of fibrous tissue and fibrous tissue bands in the uterine cavity wall, resulting in scar-free repair obstacles (Kou et al., 2020b). It has been clinically confirmed that the incidence of IUA in pregnancy accounts for about 15%–20%, which is a possible complication after abortion, and its risk increases with the number of abortions (Hooker et al., 2014). Repeated abortions and curettage are considered risk factors for adhesion formation (Hooker et al., 2014; Hooker et al., 2021).

The endometrium is composed of an upper functional layer and a lower basal layer. The functional layer falls off during menstruation and after childbirth, whereas the lower basal layer produces a new functional layer that maintains endometrial integrity (Owusu-Akyaw et al., 2019; Lv et al., 2021). The transformation of epithelial and mesenchymal cells is called epithelial-mesenchymal transformation (EMT) (Crha et al., 2019; Owusu-Akyaw et al., 2019). Type 2 EMT is associated with wound healing, tissue regeneration, and fibrosis (Marconi et al., 2021). Studies have shown that transforming growth factor beta-1 (TGF-β1), Wnt, Snail, Hippo, and other signal transduction pathways are involved in EMT, which may lead to an imbalance in this process by regulating the corresponding target molecules, thus aggravating the formation of IUA (Zhu et al., 2019; Cao et al., 2020).

Currently, the treatment of IUA mainly includes TCRA, physical barriers, drugs to assist endometrial growth, tissue membrane transplantation, and MSCs transplantation (Kou et al., 2020b; Chen et al., 2021). MSCs transplantation has broad prospects, such as bone marrow mesenchymal stem cells (BMSCs), umbilical cord mesenchymal stem cells (UC-MSCs), adipose-derived mesenchymal stem cells (ADMSCs), human amniotic mesenchymal stromal cells (hAMSCs), and endometrial mesenchymal stem cells (eMSCs), which are used in soft- and hard-tissue regeneration engineering (Benor et al., 2020; Rungsiwiwut et al., 2021; Gharibeh et al., 2022). However, the potential tumorigenicity, teratogenicity, inconvenience of transport and storage, and viability of MSC-based therapies have affected the clinical application (Benor et al., 2020).

Extracellular vesicles (EVs), also known as exosomes, have a diameter of 30–150 nm and are frequently found in various bodily fluids, including blood, urine, saliva, and breast milk (Kalluri and LeBleu, 2020). EVs can be divided into different types based on the mechanism of vesicle production, biophysical or biochemical properties, and receptor composition. The most common types include exosomes, microvesicles and apoptotic bodies. Under normal or pathological conditions, exosomes containing a series of bioactive molecules such as lipids, proteins, and nucleic acids (mRNA, miRNA, and DNA), can regulate the characteristics of target cells (Kalluri and LeBleu, 2020). MSC-derived exosomes are essential for intercellular communication (Bian et al., 2022), which can reduce the defects of MSCs therapy as a cell-free therapy, but also possess certain limitations that they can be removed from the application sites quickly and only exist for a short period of time. Thus, exosomes require a drug delivery system to prolong the treatment time (Zhou et al., 2022). At present, several drug delivery carriers have been reported, including intrauterine devices (IUDs) or Foley balloon catheters, hydrogels, collagen scaffolds, cell sheet engineering, and 3D printing scaffolds, which reduce the frequency of intrauterine administration to avoid repeated intrauterine operations that aggravate the formation of adhesions (Kou et al., 2020b; Ma et al., 2021). Therefore, tissue engineering technology has broad application prospects as a drug delivery system that can help repair damaged tissues by creating a microenvironment similar to the extracellular matrix (ECM).

**FIGURE 1:**
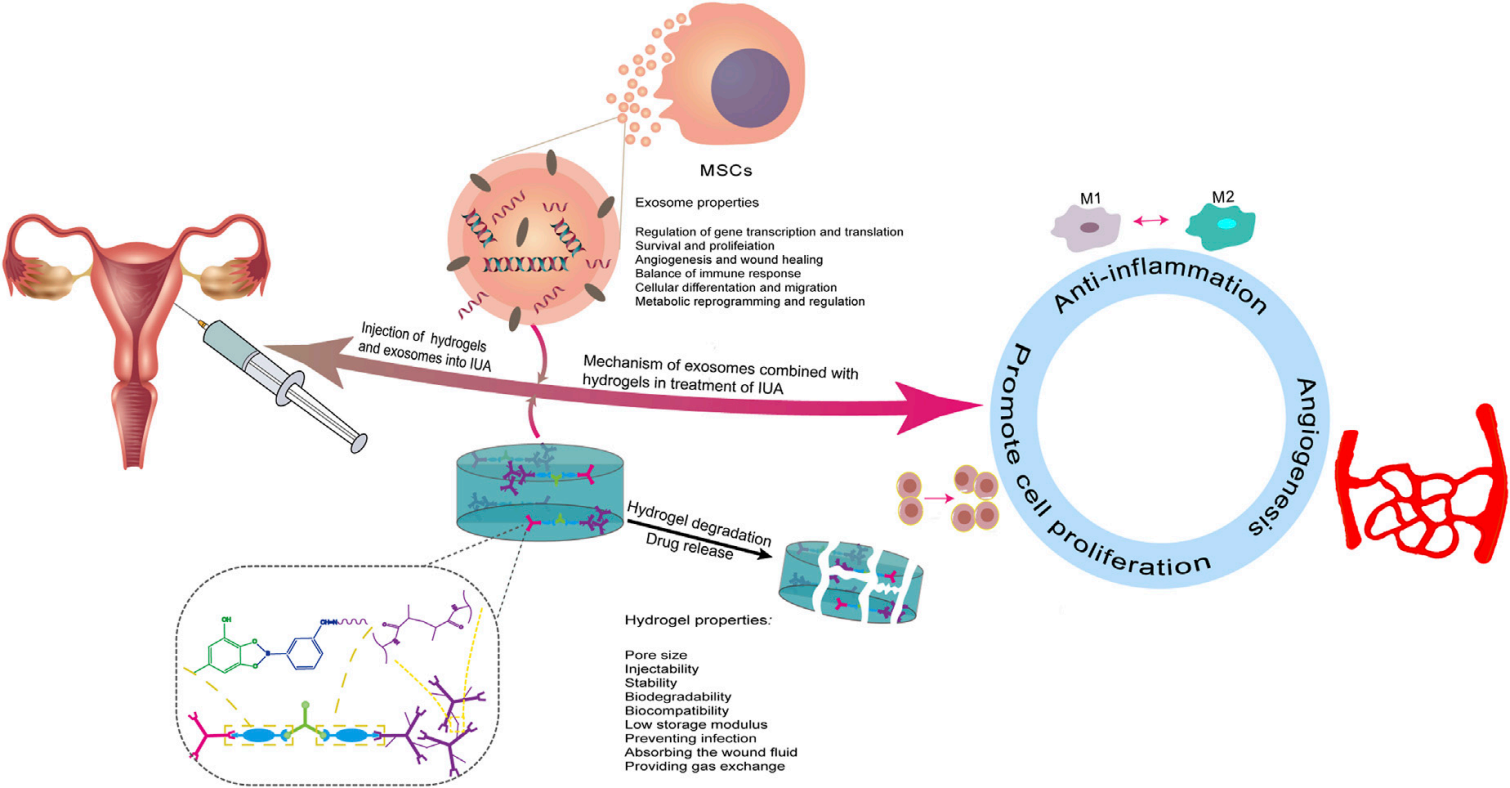
Schematic description of an overview of exosomes and hydrogel in the treatment of IUA. Exosomes are loaded on hydrogels and the mixture is injected into the uterine cavity to continuously release exosomes at the injured site to play a therapeutic role. The potential mechanisms of treatment in IUA are described: (a) anti-inflammatory, (b) promotion of angiogenesis, and (c) promotion of endometrial cell proliferation.

In general, the aqueous solution containing EVs can be injected directly into the circulatory system to enhance tissue repair, but it is difficult to retain the free EVs in the target area, thus limiting their full functions. In contrast, biomaterial-based release systems for EVs enable precise repair of damaged tissue sites in a dose and time-dependent manner that is under control (Zou et al., 2023). However, not all biomaterials can successfully carry EVs. Studies have shown that chemical, alkaline, thermal, and photo-crosslinking can enhance the binding between EVs and materials. To extend the duration of the effect time of exosomes on the injured site, exosomes and hydrogel can be introduced into the uterine cavity by constructing a hydrogel stent, which can achieve the purpose of treating IUA and improve the subsequent pregnancy rate (Figure 1). However, owing to periodic changes in the endometrium, the demand for optimal drug release must be met before the next menstruation, which requires a higher hydrogel requirement, including internal pore size, biocompatibility, and degradation time.

## 2 Pathogenic factors associated with IUA

The essence of IUA is endometrial fibrosis caused by trauma or infection. The normal matrix and epithelium are replaced by non-vascular fibrous tissue, spindle myofibroblasts, and inactive cubic columnar glands. As myofibroblasts are the primary producers of ECM, excessive buildup of ECM outside the cells exacerbates the development of endometrial fibrosis (Wei et al., 2020). Upon infection, inflammatory factors damage endometrial stem cells and limit their ability to repair the uterine cavity (Puente Gonzalo et al., 2021). This pathological change involves a variety of molecular mechanisms such as EMT (Hu M. et al., 2020; Cao et al., 2020; Yuan L. et al., 2022) and an imbalance in immune system regulation (Marofi et al., 2021).

EMT is an indispensable morphological process in the development and multi-function regulation of MSCs, but it is also activated in pathological environment such as cancer and fibrosis (Grant and Kyprianou, 2013). The dynamic transformation of EMT is jointly modified by the transcription, post-transcription, translation, and post-translational regulation of multiple signaling pathways (Kanlaya et al., 2022). Epithelial cells acquire a mesenchymal phenotype via EMT, which is required for tissue regeneration and embryonic development. This results in the loss of the epithelial marker (E-cadherin) and an increase in the mesenchymal marker (N-cadherin) (Grant and Kyprianou, 2013). The TGF-β1, Wnt, Snail, and Hippo pathways are interrelated and participate in the EMT.

Simultaneously, IUA formation may also be regulated by the immune system, which involves a dynamic balance between M1 and M2 macrophages in anti-inflammation and pro-inflammation (Kwak et al., 2022). As we know, the periodic stripping and regeneration of the endometrium is a dynamic process (Weimar et al., 2013). Unlike general damage repair, it does not result in scar formation. The damage itself involves the repair of the immune system. As the first barrier, macrophages perform irreplaceable functions during emergencies (Boniakowski et al., 2017). At the initial stage, M1 macrophages contribute to the secretion of pro-inflammatory cytokines such as tumor necrosis factor-α (TNF-α), interleukin (IL)-1α (IL-1α), IL-1β, and IL-6, which in turn accelerate the polarization of M2 macrophages and secrete anti-inflammatory cytokines such as IL-10, TGF-β1, and vascular endothelial-derived growth factor (VEGF) (Hu et al., 2018; Hu J. et al., 2020; Arabpour et al., 2021). Macrophage dysfunction can lead to fibrosis and inflammation (Wu et al., 2018). Abnormal activation of fibrosis is related to the formation of new blood vessels, which are vital for the normal uterine menstrual period (Song et al., 2021).

## 3 Current treatment strategies for IUA

### 3.1 Transcervical adhesion electroreception (TCRA)

Hysteroscopy is divided into diagnostic and surgical hysteroscopy for gynecological diagnosis and treatment. When hysteroscopic surgery is performed in patients with IUA, secondary damage to the uterine cavity caused by the use of surgical instruments cannot be ignored (Bosteels et al., 2015). Monopolar and bipolar instrumentation may lead to electrical damage to the uterine cavity and adjacent organs, such as the bladder and rectum, due to the proximity of the uterus. Thus, timely treatment must be taken (Cholkeri-Singh and Sasaki, 2016). For patients with pregnancy needs, a second pregnancy after hysteroscopic surgery increases the risk of preterm birth, intrauterine growth delay, and prenatal death (Bradley, 2002).

Before the introduction of hysteroscopy, blind dilation and curettage (D&C) were the first choices; however, the risk of uterine perforation caused by D&C has increased (Conforti et al., 2013). With advancements in surgical techniques, TCRA has become the gold standard for the treatment of IUA. Cutting off the fiber bridge between adherent uteri can restore the normal function of the uterine cavity, prevent recurrence, and promote endometrial regeneration. However, the recurrence rate of moderate and severe adhesions is very high (Warembourg et al., 2015; Dreisler and Kjer, 2019). MF et al. obtained a 95% recurrence rate of IUA by observing recovery of the endometrial cavity in patients with AS after TCRA (Salazar et al., 2017). After TCRA treatment, menstruation recovery, improvement in adhesion score, and pregnancy are key factors in evaluating the success of the operation (Khan and Goldberg, 2018). The purpose of IUA treatment is to release adhesions and prevent recurrence; therefore, prevention of adhesion reformation is the key to successful treatment.

### 3.2 Estrogen

The endometrium is histologically divided into functional and basal layers, with a thick and spongy layer covering approximately two-thirds of the surface. These layers are collectively referred to as functional layers and are influenced by ovarian sex hormones. The basal layer comprises one-third of the endometrium near the myometrium, which is unaffected by ovarian sex hormones and does not change periodically (Hapangama et al., 2015; Long and Colson, 2021). During human menstruation, only the functional layer of the endometrium facing the uterine cavity falls off, whereas the basal layer facing the myometrium remains unaffected by hormonal changes. After menstruation, estrogen secreted by the ovary stimulates the proliferation of the endometrial basal layer to reconstruct the exfoliated functional layer in the proliferative or follicular phases. If the damage reaches the basal layer, it is usually irreversible because of a decrease in the estrogen receptor, accompanied by the formation of fibrosis (Yu et al., 2022). The endometrium falls off and proliferates periodically under the action of estrogen. Intrinsic stem or progenitor cells in the endometrium develop new activities, differentiating into epithelial cells and stromal cells and promoting endometrial reconstruction for blastocyst implantation (Cen et al., 2022).

As a hormone commonly used to assist endometrial growth, estrogen is widely used to treat IUA before and after surgery (Johary et al., 2014). According to one study, preoperative estrogen application can reduce the preoperative American Fertility Society (AFS) score, which is the criteria for evaluating the severity of uterine adhesions in women, and the number of operations, and improve rates of fertility (Zhang L. et al., 2019). However, according to other clinical studies, the incidence or severity of adhesion reformation does not seem to be reduced by estrogen after surgery, regardless of the severity of pre-existing IUA. There was no difference between estrogen treatment at the third hysteroscopy and the postoperative adhesion reconstruction rate and AFS score (Yang et al., 2022). Compared with the use of estrogen alone after surgery, combined medication after surgery plays a more significant role in promoting endometrial repair and pregnancy in IUA patients, such as aspirin combined with estrogen medication (Chi et al., 2018).

### 3.3 Other treatment strategies in IUA

The use of IUDs is a safe and effective method of contraception that is used clinically as a physical barrier to prevent adhesion after uterine procedures (Long and Colson, 2021). According to a meta-analysis, IUDs improve outcomes for IUA patients, but for best results, especially in patients with moderate to severe IUA, they must be used in conjunction with other adjuvant therapies (Salma et al., 2014). In the combination treatment, IUDs combined with Foley balloon catheter has achieved good results in preventing adhesion formation and preserving the newly separated uterine cavity walls for later endometrial regeneration (Huang X. W. et al., 2020).

Platelets are anucleated cell fragments released by megakaryocytes (2∼3 μm diameter). Under normal physiological conditions, factors in platelet granules coordinate with local cells to promote tissue repair (Bos-Mikich et al., 2018; Scully et al., 2018). Platelet-rich plasma (PRP) is widely known for its ability to enhance the endometrial environment, secreting growth factors and other cytokines that activate fibroblasts and recruit leukocytes to the injury site, inducing and regulating the proliferation and migration of other cell types involved in uterine tissue repair, and influencing the reactivity of vascular and other blood cells in angiogenesis and inflammation, enhancing the endometrial environment, as demonstrated in animal studies (Goncalves et al., 2020). Jang et al. (2017) studied the therapeutic effects of PRP in damaged endometria using an experimental model of ethanol-induced injury in rats. They found that intrauterine application of autologous PRP promoted and expedited endometrial regeneration and reduced fibrosis. Extracorporeal shock wave therapy and PRP have also been used in a rat model of IUA during preventative and therapeutic stages (Cheng et al., 2022). In a clinical trial on the treatment of refractory endometrium with PRP as an adjuvant for endometrial preparation, 19 patients with refractory endometrium were recruited and PRP was introduced into the uterine cavity. It was found that endometrial thickness increased, and positive pregnancy tests, clinical pregnancy, and live births were obtained (Zhang S. et al., 2019).

MSCs are pluripotent cells that can self-renew and differentiate into many cell types, which are essential for tissue repair and regenerative therapy (Fu et al., 2019). MSCs can differentiate into cells of mesodermal origin, which occurs in the embryo from which the endometrium originates. MSCs are commonly derived from the bone marrow; however, recent studies have shown that MSCs can be successfully isolated from adipose tissue, umbilical cord (Malhotra et al., 2016), menstrual blood, and other tissues, demonstrating the initial safety and effectiveness profiles in the resumption of menstruation, fertility outcomes, and endometrial regeneration (Benor et al., 2020). Two types of MSCs have been reported to have potential applications in IUA treatment, which are BMSCs and UC-MSCs. Firstly, the efficacy of BMSCs in cell therapy depends on their ability to homing and long-term implantation into the injured site. The contribution of BMSCs in promoting new angiogenesis has been confirmed in animal models, but human clinical trials are not widely applied (Zhuang et al., 2022). Previous reports showed that the number of CD34^+^, C-kit^+^, or Flk-1^+^ cells, and the markers of endothelial lineage cells had increased in the wound healing process after the treatment of BMSCs conditioned medium (Wu et al., 2007). By analyzing the expression of a large number of angiogenic cytokines, such as VEGF-A, IGF-1, PDGF-BB, and Ang-1 in a conditioned medium of BMSCs, it was found that BMSCs might promote the recruitment of endothelial cells (ECs) and endothelial progenitor cells (EPCs) by secreting angiogenic cytokines (Chen et al., 2008). Secondly, UC-MSCs isolated from the umbilical cord have strong self-renewal and proliferation activities. Moreover, the low immunogenicity and non-tumorigenicity features make UC-MSCs promising bio-materials that also avoid ethical issues (Song et al., 2021; Feng et al., 2022). A recent report showed that UC-MSCs could enhance endometrial cell spread and vascular revascularization while inhibiting excessive fibrosis and inflammation, thereby having the potential to repair damaged endometrium and restore fertility (Zhang et al., 2018). *In vitro* studies showed that when MSCs were co-cultured with macrophages induced by lipopolysaccharide (LPS), the number of M2 macrophages with an anti-inflammatory phenotype increased with the increased secretion of IL-10 and VEGF, suppressed production of TNF-α and IL-6 (Zhang S. et al., 2020). In another study that used the combination of UC-MSCs and collagen fibers, UC-MSCs may promote the degradation of collagen by upregulating the expression of matrix metalloproteinase 9 (MMP 9) in the system and the regeneration of endometrium and myometrium (Xu L. et al., 2017).

## 4 Exosome combined with hydrogel in the treatment of IUA

Since the current treatment strategy is insufficient to improve the cure rate of IUA, it is imperative to develop new approaches to improve the intrauterine environment. Exosomes promote tissue repair and regeneration and play a functional role in paracrine signal transduction, similar to MSCs transplantation in the treatment of diseases (Xing et al., 2021; Ju et al., 2023). Exosomes have broad application prospects as a medium for intercellular communication; however, some challenges still remain. Simple exosome injections have insufficient interaction time to produce long-lasting effects and the unique shape of the uterus prevents the liquid mixture from remaining in the uterine cavity. Solving these problems requires an exosome carrier that is suitable for uterine cavity morphology. Conventional exosome carriers have certain drawbacks, such as low biocompatibility, immunogenicity, and mechanical instability. IUDs or Foley balloon catheters have a low biocompatibility for not fitting the uterine cavity well, amnion membrane may induce an immune response for its limited sources, the mechanical strength of ECM and cell sheet is too weak to keep their shape and easy to degrade (Kou et al., 2020a; You et al., 2022). Therapeutic cell delivery strategies have been developed to prevent and treat IUAs, including IUD, hydrogel, collagen scaffold, carrier-free cells, poloxamers, and decellularized extracellular matrix (dECM) (Kou et al., 2020b; Li X. et al., 2021). Currently, most research on the combined application of exosome and hydrogels in the treatment of IUA is still in the cell experimental stage and animal experimental stage, which has not entered clinical trials. However, reviewing these studies may provide potential applications in the treatment of IUA in clinics in the future.

### 4.1 Biological characteristics of MSCs-derived exosomes

Most cells produce exosomes for communication under physiological and pathological conditions (Yang et al., 2019). As a bridge for information exchange between cells, exosomes are secreted from cells through exocytosis, and a large number of biological signals such as lipids, proteins, and nucleic acids are transmitted to receptor cells (He et al., 2018). Therefore, exosomes present in biological fluids can be used for diagnostic and therapeutic properties (Mori et al., 2019; Zhang Y. et al., 2020; Isaac et al., 2021). Although many mechanisms of exosome biosynthesis have been identified, the most important is the endosomal sorting complex required for the transport (ESCRT). The ESCRT system consists of four components with auxiliary proteins that work synergistically and stepwisely to form the multivesicular bodies (Liang et al., 2021).

MSC-Exos play an important role in tissue damage repair and regeneration. Exosomes can be isolated from MSCs and the number of exosomes can be controlled at the injured site. Owing to their unique plasma membrane structure, exosomes maintain their biological activity at low temperatures, embed drug molecules, and deliver them to specific sites. The internal mRNA and miRNA form the basis of exosome function (Kou et al., 2022). By isolating exosomes from ADMSCs (ADMSC-Exos), BMSCs (BMSC-Exos), and UC-MSCs (UC-MSC-Exos), Hoang et al. (2020) found that all exosomes from these three sources promoted the proliferation and migration of target cells, tissue cells at the site of injury, and the effects of MSC-Exos on target cells varied in a dose-dependent manner. However, further research is needed to study the mechanism of MSC-Exos in the repair of tissue damage processes, particularly the relationship between the source and dose of exosomes and their target cells.

As the most commonly used MSCs, BMSCs have received considerable attention owing to their extensive origin, low immunogenicity, and lack of ethical controversy (Das et al., 2019). MSCs have been shown to increase endometrial cell spread and vascular revascularization while limiting excessive fibrosis and inflammation in recent research, healing damaged endometrium, and restoring fertility (Zhang et al., 2018). Subsequently, Nakao et al. (2021) revealed that exosomes isolated from MSCs promoted M2 macrophage polarization via the Wnt5a-mediated RANKL pathway. In another study on the combination of MSCs and collagen fibers, it was found that MSCs promoted the degradation of collagen by upregulating the expression of MMP9 in the system and increased the regeneration of the endometrium and myometrium (Xu L. et al., 2017).

### 4.2 Application of exosomes in the treatment of IUA

Although great progress has been made with exosomes in wound healing, their long-term treatment results in patients with IUA remain unclear (Bian et al., 2022). MicroRNAs (miRNAs) are regulatory non-coding RNA secreted by exosomes under the influence of the external environment and play a role in signal transmission (Lu et al., 2020; Li B. et al., 2021). Here, we have listed some miRNAs contributing to injury treatment and summarized their possible therapeutic mechanisms (Table 1). MSC-Exos have three functions in the treatment of IUA (Wu et al., 2018; Marofi et al., 2021; Bian et al., 2022). First, MSCs directly contact each other through exosomes by reducing the production of proinflammatory factors and increasing the expression of anti-inflammatory factors. This process involves macrophage polarization. Second, exosomes isolated from MSCs significantly reversed endometrial fibrosis through a series of signal transduction pathways such as TGF-β, Wnt, Snail, and Hippo. Finally, modification of MSCs to produce specific exosomes can enhance angiogenesis.

**TABLE 1:** Summary of the research methods and possible mechanisms of exosomes and miRNAs from different sources in injury treatment.

MiRNAs	Methods	Mechanisms	References
miR223-3p	designing the construct of UC-MSC-Exos and collagen scaffolds, and analyzing RNA sequences in exosomes	the polarization of M2 macrophages was promoted, which reduced the pro-inflammatory response and increased the anti-inflammatory response	Xin et al. (2020)
miR26a-5p	introducing miRNA into exosomes and combining it with hydrogel	the key molecule APY29 regulated macrophage polarization and miRNA played a vital role in anti-inflammatory, angiogenesis, and immune regulation	Mi et al. (2022)
miR205-5p	combination of silk hydrogel and hypoxia-pretreated BMSCs-Exos	the proliferation, migration, and anabolism of target cells was enhanced, and the inflammation was inhibited	Shen et al. (2022)
miR-31-5p	using milk-derived exosomes as a novel system for miR-31-5p delivery	ECs’ functions were dramatically improved *in vitro*, together with the promotion of angiogenesis and wound healing *in vivo*	Yan et al. (2022)
miR221-3p	extracting exosomes from BMSCs to explore the effect on HUVEC *in vitro* experiments, and exosomes were injected into the injured site *in vivo* to detect wound healing	the biological function of ECs was strengthened via AKT/eNOS pathway by upregulating the miR-221-3p	Yu et al. (2020)
miR216a-5p	comparing the effects of the hypoxic preconditioning group and normoxic exosome group on injury treatment *in vitro* and *in vivo*	after hypoxia pretreatment, miR-216a-5p was enriched in exosomes, and TLR4/NF-κB/PI3K/AKT signaling cascades may be involved in the modulation of microglial polarization	Liu et al. (2020b)

Exosomes participate in immune regulation by regulating macrophage polarization, which is beneficial for angiogenesis, (Huang Y. et al., 2020; Li R. et al., 2022). He et al. (2019) found that inhibiting MSC-Exos decreased the number of M2 macrophages compared with normal MSC-Exos expression system in a co-culture system *in vitro* and *in vivo*. It indicates that MSCs could establish a relationship with macrophages by secreting exosomes and inducing polarization of M2 macrophages. MSC-Exos can accelerate the inflammatory process by decreasing the expression of pro-inflammatory factors and increasing the expression of anti-inflammatory factors at the site of injury (Guan et al., 2022). This promotes conversion between M1 and M2 macrophages, which accelerates endothelial cell regeneration and blood vessel formation (Lv et al., 2022). Ti et al. (2015) activated the expression of anti-inflammatory factors in UC-MSCs by inducing UC-MSCs with LPS and extracting exosomes from LPS pre-UC-MSCs (LPS pre-Exo). miRNA microarray analysis was used to obtain the miRNA expression profiles of LPS pre-Exo cells. Compared with exosomes isolated from UC-MSCs without the induction of LPS (LPS un-Exo), there were 40 miRNAs with significantly different expression levels. Using a hierarchical clustering graph (heat map) generated by TIGR multiple experimental viewers, five miRNAs were found to exist only in LPS pre-Exo. Analysis of the database revealed that Let-7b had the highest expression level among the five unique miRNAs of LPS pre-Exo. Let-7b regulates macrophage polarization via the TLR4/NF-κB/STAT3/AKT signaling pathway. Using LPS pre-Exo in the skin injury model of diabetic rats, it was found that LPS pre-exo markedly stimulated the emergence of new small capillaries and wound healing and increased M2 macrophage expression levels while decreasing M1 macrophage expression. Zhang et al. (2021c) found that UC-MSC-Exos promoted the proliferation, migration, angiogenesis, and differentiation of EPCs *in vitro*. Importantly, mechanistic research revealed that exosomal miR-21 secreted by UC-MSCs was a potential intercellular messenger that stimulated the NOTCH1/DLL4 pathway to promote EPCs’ function. Li X. T. et al. (2020) isolated exosomes from BMSCs to study their effects on the proliferation of microvascular ECs in rats. They found that BMSCs and BMSC-Exos treatment promoted the proliferation of ECs, but there was no significant difference between the two groups (*p* > 0.05), indicating that exosomes can be used as an alternative therapy for BMSCs in promoting angiogenesis. During endometrial injury, the persistent presence of M1 macrophages stimulates fibroblasts to secrete excessive ECM, which leads to IUA formation. According to another study, local injection of exosomes reduced pro-inflammatory factors secreted by macrophages, thereby inhibiting endometrial cell apoptosis and promoting tissue repair (Shi et al., 2020).

Exosomes have been implicated in uterine cavity injury with a role in anti-endometrial fibrosis, and the mechanism is mainly related to EMT (Wang H. et al., 2022). According to a previous study, injection of MSC-Exos into the myometrium of animals significantly increased the expression of CK19 and decreased VIM expression. Compared to the control group, the expression of TGF-β/Smad signaling pathway-related factors decreased, and after the fourth week of treatment, there was no significant difference between the exosome therapy and sham control groups (*p* > 0.05). This demonstrated that the TGF-β/Smad signal transduction pathway participated in the EMT process, and exosome therapy reversed the development of this transversion (Yao et al., 2019). When the endometrium is damaged, the presence of excessive pro-inflammatory factors interferes with the level of calcium ions, and the latter abnormality causes mitochondrial dysfunction, resulting in endoplasmic reticulum misfolding or accumulation of unfolded proteins, triggering endoplasmic reticulum stress (ERS), eventually leading to cell death, and IUA also occurs under this condition (Han et al., 2018; Liu J. et al., 2022). Further research showed that ERS may participated in the EMT process and promoted the occurrence of IUA through the TGF-β/SMAD pathway (Bao et al., 2023).

Surface modifications, including covalent and non-covalent modifications, give exosomes the characteristics to function at specific sites, and this can also be used as a carrier for carrying therapeutic drugs, most of which are used in cancer treatment (Salunkhe et al., 2020). Although there are no direct studies on the surface modification of exosomes in the treatment of IUA so far, some existing research provides insight into this field. A study by Tamura et al. (2017) extracted exosomes from MSCs and modified them with cationized pullulan to target cell surface glycoprotein receptors. These modified exosomes could better target the damaged site, which may provide certain potential application prospects in IUA. Recent studies have shown that genetic modifications remarkably enhanced the regeneration and angiogenic activity of MSC-Exos. Zhu et al. used an adenovirus vector to overexpress Cardiotropin-1 (CTF1), a cytokine in the IL-6 family (Martinez-Martinez et al., 2019), transfected it into BMSCs, and extracted exosomes (C-BMSCs-Exos) from BMSC overexpressing CTF1. Compared to control BMSCs (BMSC-Exos), C-BMSC-Exos significantly promoted the proliferation, migration, and tube formation of human umbilical vein endothelial cells (HUVECs) *in vitro*, promoted endometrial regeneration and restored fertility in a rat endometrial injury model (Zhu et al., 2022). Li X. et al. (2020) transfected BMSCs with a lentivirus encoded by CXCR4 and isolated exosomes. CXCR4 in BMSCs can further promote the proliferation and tube formation of endothelial angiogenesis. Studies have shown that exosomes overexpressing hypoxia-inducible factor 1-α (HIF-1α) significantly promote angiogenesis in ischemic heart disease, but research on endometrial injury has not yet been conducted (Sun et al., 2020). HIF-1α plays a crucial role in immune and inflammatory processes by regulating the expression of related genes (McGettrick and O'Neill, 2020). Exosomes isolated from MSCs after hypoxic preconditioning showed better results in angiogenesis, proliferation, and migration compared to those secreted under normoxic conditions, and further experiments confirmed the relationship between exosomal miR-126 and HIF-1α (Liu et al., 2020a). Therefore, these reported molecules may have potential research value in models of uterine cavity injury that need further study.

### 4.3 Hydrogels’ bio-characteristics and application in the treatment of IUA

The natural ECM, which has been widely used as a support for stem cell adhesion, migration, differentiation, and proliferation (Xin et al., 2020), is mainly composed of two major macromolecules proteoglycan and fibrin. Owing to its unique buffering, hydration, binding, and load-bearing properties, proteoglycan fills most of the gaps in the tissue in the form of a hydrogel (Padhi and Nain, 2020). Similar to the ECM, hydrogels maintain structural integrity through physical and chemical actions, which make them cell carriers and conducive to creating a microenvironment suitable for cell growth (Wang and Wang, 2021; Jin et al., 2022). Hydrogels are highly crosslinked macromolecular hydrated networks. This soft and humid material decomposes while performing its functions owing to its constant water absorption and loss characteristics (Cheng et al., 2021). Generally, they are defined as polymer systems that can expand in water, retain at least 20% water in their 3D structures, and are insoluble in water (Sharma and Tiwari, 2020). Natural and biosynthetic hydrogels with biological activities and functions can be used not only as biomaterials to prevent IUA but also as carriers for hormone drugs, growth factors, and MSCs to maximize benefits for the recovery of the uterine cavity (Wang J. et al., 2021) (Table 2). Thus, good injectability, stability, biodegradability, low storage modulus, and excellent biocompatibility must be considered for using such biomaterials in endometrial injury (Wang et al., 2020; Lopez-Martinez et al., 2021; Wang L. et al., 2022). Additionally, some hydrogels have the advantages of preventing infection, absorbing wound fluid, and providing gas exchange (Mao et al., 2017). Developing injectable self-healing adhesive hydrogels with built-in antibacterial activity for drug administration is essential and encouraging for promoting healing after TCRA.

**TABLE 2:** Summary of biomaterial-based hydrogels as a delivery system to deliver drugs in the repair of endometrial injury.

Drugs	Biomaterials	Models	References
Estrogen	PHEMA hydrogel	Rat	Yu et al. (2022)
β-estradiol	Heparin-Poloxamer Hydrogel	Rat	Zhang et al. (2017); Zhang et al. (2020b)
β-estradiol	Aloe/poloxamer hydrogel	Rat	Yao et al. (2020)
Vitamin C	hydrogel Pluronic F-127	Rat	Yang et al. (2017)
KGF	Temperature-sensitive heparin-modified poloxamer hydrogel	Rat	Xu et al. (2017b)
G-CSF	3D-printed hydrogel scaffold	Rat	Wen et al. (2022)
KGF	heparin-modified poloxamer and EPL	Rat	Xu et al. (2017a)
bFGF	collagen scaffolds	Human	Jiang et al. (2019)
bFGF	GelMA-Na-alginate-loaded porous scaffold	Rat	Cai et al. (2019)
GFs	EndoECM	Rat	Lopez-Martinez et al. (2021)
IGF-1C	chitosan hydrogel	Rat	Li et al. (2020a)
SDF-1α	chitosan-heparin hydrogel	Rat	Wenbo et al. (2020)
UC-MSCs	collagen Scaffold	Rat	Xin et al. (2019); Liu et al. (2020c)
UC-MSCs	collagen scaffold	Human	Cao et al. (2018); Zhang et al. (2021b)
UC-MSCs	gelatin/sericin hydrogel	Rat	Chen et al. (2023a)
UC-MSCs	Pluronic F127/hyaluronic acid hydrogel	Rat	Hu et al. (2022)
hiMSCs	3D-printed hydrogel scaffold	Rat	Ji et al. (2020a)
HP-MSCs	hyaluronic acid hydrogels	Rat	Lin et al. (2022)
BMSCs	collagen scaffolds	Rat	Ding et al. (2014); Chen et al. (2022)
BMSCs	PGS scaffold	Rat	Xiao et al. (2019)
EMSCs	hyaluronic acid hydrogel	Rat	Kim et al. (2019)
EVs	hyaluronic acid Hydrogel	Rat	Liu et al. (2019)
ABs	hyaluronic acid hydrogel	Rat	Xin et al. (2022)

Recently, as a vehicle for drug delivery, hydrogels have shown great potential in endometrial regeneration to regulate the abnormal physical environment of the IUA. However, the use of bioactive hydrogels to encourage endometrial tissue regeneration is currently in the exploratory phase (Avila-Salas et al., 2019). The hydrogel used for IUA must accommodate the inverted pear-shaped uterine cavity structure and adhere to the damaged area for a certain period to prevent re-adhesion (Khayambashi et al., 2021). A meta-analysis of randomized studies found that utilizing hydrogels considerably decreased the frequency of moderate-to-severe IUA, but had no effect on mild IUA. A much higher pregnancy rate has also been linked to this finding (Liu Y. R. et al., 2022). However, two other clinical trials of the application of hydrogels seemed to produce different results that the menstrual pattern after mild to moderate IUA did not improve (Zhou et al., 2021; Guo et al., 2022). Therefore, the clinical application of hydrogels after uterine injury requires further research to guide subsequent treatments.

Interestingly, physical or chemical crosslinking can also change the mechanical properties of hydrogels such as hydrophobic interactions, hydrogen bonding, polymerization entanglement, and π–π stacking (Sharma and Tiwari, 2020; Cao et al., 2021; Cha et al., 2022). Gao et al. (2022) prepared an injectable H-HPMA hydrogel via free radical polymerization of methacrylate hyaluronic acid (HA-GMA) and N-(2-hydroxypropyl) methylacrylamide (HPMA) monomers in an aqueous solution. Many reversible hydrogen bond networks provide a strong antifouling ability. It exhibits excellent self-fusion, antifouling, and injection performances. Further studies have shown that it can repair peritoneal injury in rats and shorten peritoneal healing time. The PEBP/PEG hydrogel also suppressed the proliferation of fibroblasts through π-π accumulation by regulating the expression and interactions of TGF-β1and Muc-4, and the *in vivo* preventive effect of the PEBP/PEG hydrogel on fibrosis prevention and pregnancy-promoting effects was obvious (Wang B. et al., 2021). Lin et al. (2021) used a PDE hydrogel and exosomes isolated from adipose stem cells (ADSC-Exos) to crosslink through Ag-S coordination and designed an exosome-hydrogel system with microenvironment-protective properties that can promote endometrial regeneration and fertility recovery. *In vitro*, this system was used to measure the tubulogenesis, migration, and proliferation of HUVECs, and was found to enhance their angiogenic ability. In a rat model of endometrial injury, by assessing intrauterine embryo implantation rate at 18 days gestation, ADSC-Exo hydrogel groups were found to promote endometrial regeneration compared to the sham operated control group.

Certain hydrogels exhibit sol-gel transitions in response to environmental factors, such as temperature, pH, and light (Xiao et al., 2021). Under physiological conditions, the pH of the vagina and cervix is slightly acidic, and when pathological factors affect the reproductive system, its microecology is destroyed. Therefore, the application of pH-responsive hydrogels in the treatment of uterine injury is crucial. Hydrogels with unique thermosensitive properties have also been developed, which exist in the form of liquids at low temperatures and semi-solid gels at high temperatures, such as poloxamers and Pluronic F-127, which also absorb secretions on the wound surface to maintain a moist and sufficient healing environment (Yang et al., 2020). However, it is difficult for these biodegradable hydrogels to maintain their shape in complex organisms, especially when wounds are very large. Therefore, hydrogels can be reasonably combined with scaffolds to achieve good compatibility and mechanical properties (Ji X. et al., 2020). 3D-printed poly (ε-caprolactone) scaffold was designed to form a composite scaffold, which combines an injectable thermosensitive chitosan hydrogel with a scaffold that has a porous structure and good mechanical strength, thus providing strong support for the hydrogel. The hybrid scaffold provides an environment for MSCs to grow while allowing new tissues to grow (Dong et al., 2017).

In the meanwhile, the development of 3D printing technology in tissue engineering has resulted in significant prospects. Hydrogels have gradually emerged from the public perspective as raw materials for printing. It must have the following characteristics. Firstly, the printability and crosslink ability require hydrogel prepolymers to have a certain surface tension to maintain specific morphological structures. Secondly, they should have mechanical properties, including strain, shear stress, compressive modulus, and mass swelling ratio, which together create a stable environment for cell adhesion, proliferation, and differentiation. Finally, biocompatibility and controllability of degradation mean that no exclusion reaction occurs with the host in a specific environment, and hydrogels need a degradation time similar to that of the ECM and have no toxic effects on cells (Mandrycky et al., 2016; Shen et al., 2022). The porous structure of the hydrogel can carry drugs, whereas the printed 3D stent hydrogel can adapt to the complex intrauterine environment to provide support, which combined together to promote injury recovery (Abdollahiyan et al., 2020). Some related studies have made some progress in this field. Liu et al. (2021) used phenole-functionalized chitosan and dibenzaldehyde-terminated telechelic poly to form a self-healing hydrogel with a fast-gelling rate, good self-healing ability, and facile printability for 3D printing. The hMSCs were then embedded in the hydrogel and the cytotoxicity of the hydrogel was evaluated by live/dead cell staining. hMSCs proliferation was evident after 24 h of cultivation. An *in vitro* study used MSCs to co-culture with alginate-hyaluronic acid hydrogel-based two-layer endometrial structure based on 3D bioprinting technology, and the effects of the material on the proliferation and toxicity of L929 cells were evaluated by live-dead assays and CCK-8 assays. It was found that the co-culture system could improve the biological activity of the cells by promoting endometrial functional regeneration and postoperative pregnancy reproductive outcome in a partial full-thickness uterine excision rat model (Nie et al., 2023). In recent years, several novel hydrogels, such as silk fibroin hydrogels (Hong et al., 2020), nano-polysaccharide self-healing hydrogels (Heidarian et al., 2022), heterogeneous hybrid hydrogels (Xu et al., 2022), and methylated hydroxynic acid (MeHA) with gelatin methacryloyl (GelMA) (Bittner et al., 2021) have been used as 3D-printed materials to repair tissue damage and have achieved certain results in preclinical research.

### 4.4 Combined application of exosomes and hydrogels in the treatment of IUA

**FIGURE 2:**
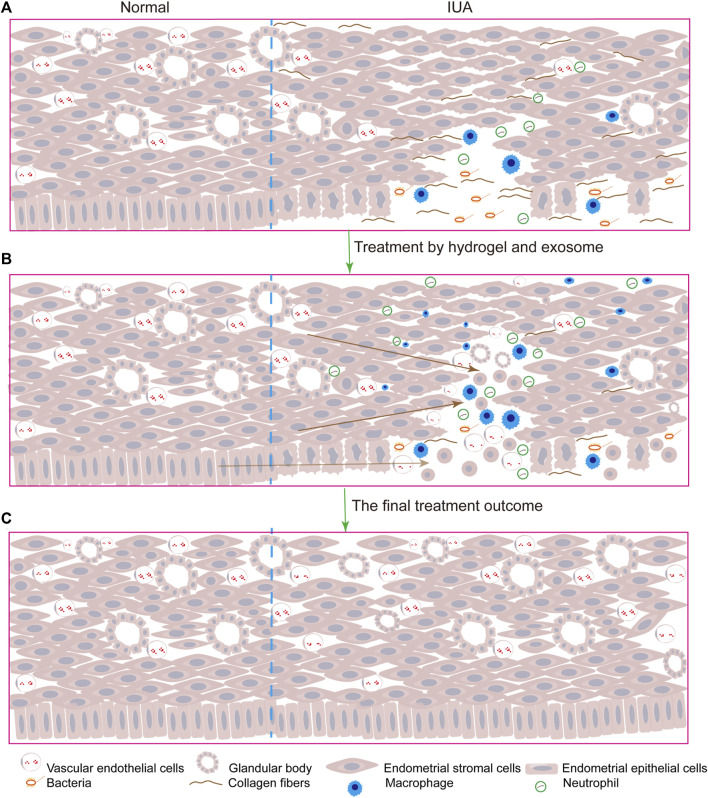
The model diagram of exosomes combined with hydrogels therapy for IUA is as follows: **(A)**. After the endometrial and stroma were damaged, inflammatory cells infiltrated, collagen fibers replaced normal tissues, the number of glands and blood vessels was reduced and bacteria polluted; **(B)**. After the injection of exosomes and hydrogels, the uterus began to repair, and endometrial progenitor cells migrated to the injured site and differentiated into epithelial cells and stromal cells, and inflammatory cells played an anti-inflammatory role to promote tissue repair and increase angiogenesis; **(C)**. Uterine cavity returned to normal.

The delivery of a large number of exosomes to the injury site is crucial for IUA repair. Different types of biomaterials such as hydrogels, films, and foams can be used as delivery systems for exosomes. They are composed of natural or synthetic polymers, such as alginate, chitosan, collagen, and gelatin. Alginate has greater chemical flexibility compared to other biocompatible degradable materials and more closely mimics the physical properties of mammalian ECM, but its mechanical strength is insufficient to allow physical loading, and degradation is relatively rapid. Therefore, the combined use of biomaterials to overcome the limitations of a single material is essential. In contrast to other tissue injuries, owing to the special morphology of the uterine cavity, exosomes injected in the origin injury sites would still flow out of the body along the vagina that impair their therapeutic functions. Although many drug delivery systems have emerged in recent years, compared to clinical practice, the emergence of hydrogels has great application prospects. The therapeutic advantage of hydrogels is that they continuously release exosomes at the injured site, thus maintaining a high local drug concentration for a long period and reducing repeated drug administration in clinical practice. Therefore, they may become a promising drug based on exosomes for treating IUA (Pan et al., 2020; Wang J. et al., 2022). Traditional hydrogels are prone to changes in their mechanical properties under external pressure in a complex physiological environment due to their incomplete compatibility with the damaged site. However, some hydrogels have excellent self-healing properties, which make them better to make a difference at the site of injury. In one study, the use of self-healing hydrogels to carry exosomes resulted in better therapeutic outcomes than treatment with exosomes alone. The HUVECs mobility and microvascular density were significantly different between the two groups (*p* < 0.05). H&E and Masson’s Trichrome Staining revealed inconspicuous tissue immune rejection (Wang et al., 2020). Here, we summarize some applications of exosomes combined with hydrogels in damaged tissue repair (Table 3), and the model diagram of the combined exosome-hydrogel therapy mechanism was illustrated in Figure 2.

**TABLE 3:** Research progress of exosomes combined with hydrogels in different diseases.

Hydrogels	Exosomes	Application direction	References
HA-based hydrogel with MnO2/FGF-2	M2-Exos overexpressing miR-223	Diabetic Wound Healing	Xiong et al. (2022)
GelMA/PEGDA	HUVECs-derived exosomes	Diabetic Wound Healing	Yuan et al. (2022b)
Polypeptide-based FHE hydrogel	ADMSCs-Exos	Diabetic Wound Healing	Wang et al. (2019)
Pluronic F127 Hydrogel	hUC-MSC-Exos	Diabetic Wound Healing	(Yang et al., 2020)
MMP-PEG smart hydrogel	ADSC-Exos	Diabetic Wound Healing	Jiang et al. (2022)
PEG hydrogels	M2-Exos	Cutaneous Wound Healing	Kwak et al. (2022)
GelMA hydrogel	MSC-Exos	Spinal Cord Repair	Han et al. (2022)
GMPE hydrogel	BMSC-Exos	Spinal Cord Repair	Fan et al. (2022)
Peptide-modified adhesive hydrogel	Hypoxia-stimulated MSCs-Exos	Spinal Cord Injury	Mu et al. (2022)
Hyaluronan-collagen hydrogel	BMSC-Exos	Traumatic Brain Injury	Liu et al. (2023b)
Hydrogel based on QCS and OST	MSC-Exos	Disc Degeneration	Guan et al. (2023)
ECM hydrogel	ADSC-Exos	Intervertebral Disc Degeneration	Xing et al. (2021)
HA hydrogel with SDF-1α	M2-Exos	Bone Regeneration	Chen et al. (2023b)
AD/CS/RSF hydrogel	BMSC-Exos	Cartilage Defect Regeneration	Zhang et al. (2021a)
HG hydrogel	ADSC-Exos	Cavernous Nerve Injury	Liu et al. (2023a)

Many studies have explored hydrogels as carriers to load cells or drugs to promote target cell proliferation and blood vessel formation. It seems that temperature-sensitive hydrogels become the first choice for carrying cells, drugs, and exosomes in order to adapt to the intrauterine environment. The combined application of hUC-MSC-Exos and Pluronic F-127 hydrogels significantly accelerated the wound closure rate and cell proliferation of HUVECs compared with the control group via the wound healing assay and CCK-8 assay (Yang et al., 2020). In another study, hADSC-Exos were embedded in a Pluronic F-127 hydrogel, and wound healing was accelerated by enhancing angiogenesis and collagen remodeling using immunostaining analysis (Zhou et al., 2022). Pluronic F-127 has been approved for human use by FDA owing to its low toxicity, biocompatibility, and thermal reversibility, and it exerts no effect on cell survival and proliferation (Yang et al., 2017). An increasing number of studies have shown that the combination of hydrogels with different therapies renders drug delivery systems more powerful. Wang et al. (2019) developed an injectable self-healing polypeptide hydrogel. The gel exhibited inherent antibacterial activity and pH-responsive long-term exosome release. Through the combined application of Pluronic F-127, oxidative hyaluronic acid, and EPL, exosomes can be better constructed in the hydrogel and released into the damaged site in a weakly acidic environment.

The use of natural or synthetic hydrogels alone still has limitations in promoting the regeneration of damaged tissues; therefore, the combination of a variety of bioactive materials has great potential for future studies. Further research has demonstrated the broad prospects of multifunctional hydrogels as drug delivery systems. A novel *in situ* injectable HA@MnO_2_/FGF-2/Exos hydrogel, which forms a protective barrier covering the wound, increases the antibacterial activity of the wound surface (Xiong et al., 2022). In addition, the use of hydrogels as materials for 3D printing results in improved mechanical properties and cell compatibility. According to a study, MeHA is widely used to make raw materials for 3D printing. Compared to traditional hydrogels, 3D-printed MeHA has better mechanical properties, swelling properties, and biocompatibility. As a delivery material carrying hMSC-Exos, it not only adapts to wound damage but also extends the action time of exosomes at the injury site (Ferroni et al., 2022). Due to the wide application of MeHA in 3D printing, researchers used MeHA and ECM photo-crosslinked at 37° as printing ink (Li C. et al., 2022), GelMA as the mixed bio-ink (Hossain Rakin et al., 2021), from which we have learned these studies demonstrate the potential value of MeHA as a 3D-printed biomaterial and its powerful ability as a drug delivery system in delivery cells and exosomes.

## 5 Conclusion and future perspectives

The treatment of IUA is mainly through surgery currently, however, IUA formation may be aggravated after surgery in patients with moderate-to-severe IUA. Therefore, appropriate adjuvant treatment is important for reducing the occurrence of re-adhesions after surgery. However, common treatment strategies include the use of hormones, PRP, hydrogel, IUDs, and Foley balloon catheters alone or in combination cannot significantly improve the recovery of patients after surgery. Although MSCs transplantation has shown obvious effects in clinical practice, the potential tumorigenicity and immunogenicity of MSCs remain controversial. Recent research reveals that MSC-Exos have the potential to serve as a bridge for information exchange in MSCs treatments, thus exosomes isolated from MSCs may solve the problems arising from MSCs therapy. Due to the special structural properties of uterine cavity, exosomes alone are hard to exist and function for a long time in the injured site, thus biocompatible carriers should be considered in the applications of exosomes.

The emergence of biocompatible hydrogels has created an external environment similar to that of the ECM for exosomes and has achieved remarkable effects on tissue damage and regeneration. Biomacromolecule hydrogels have a variety of unique properties that enable them to be used in various fields, such as biomedical, industrial, environmental, and agricultural fields. However, owing to the particularity of the uterine cavity, a 3D-printed scaffold can be set up according to the inverted pear shape of the uterine cavity to adapt to its structure in the uterine cavity. Thus, the application of hydrogels in 3D printing can be an advanced technology to load exosomes that continuously release exosomes. Despite some difficulties and challenges that remained in manufacturing, 3D printing has shown great potential in tissue engineering applications with a bright future.

According to the current research and prospects, we mainly discuss three aspects in this review, hoping to give new insights into the future treatment strategies of IUA. Firstly, by studying the pathological mechanism of IUA, it can be seen that periodic exfoliation and repair of normal endometrium is a dynamic process. Different from general injury, there is no scar formation, which involves complex physiological and pathological processes. Here we discussed the pathological mechanism of fibrosis, EMT, and inflammation. Secondly, hydrogels are not only used as a single carrier for drug transportation but also create a favorable microenvironment for tissue repair due to their excellent biocompatibility. Therefore, how to design a bioactive hydrogel that has good properties such as appropriate pore size, injectability, biodegradability, and biocompatibility should be considered in the early research design. In addition, when it comes to clinical applications, other problems such as preventing infection, absorbing the wound fluid, and providing gas exchange should also be tested. Finally, exosomes, as the bridge of information transmission in MSCs, overcome the shortcomings of MSCs and can be modified to enhance the therapeutic effect. So genetic modifications of MSC-Exos strategies are promising for the development of IUA treatment. In conclusion, the combination of biomaterials such as hydrogels with exosome delivery technology and tissue engineering technology is expected to solve the bottleneck of currently available clinical IUA treatment.

## Figures and Tables

**Table:** 

**IUA**	intrauterine adhesion
**AS**	Asherman syndrome
**TCRA**	transcervical adhesion electroreception
**MSCs**	mesenchymal stem cells
**EMT**	epithelial-mesenchymal transformation
**TGF-β1**	transforming growth factor beta 1
**BMSCs**	bone marrow mesenchymal stem cells
**UC-MSCs**	umbilical cord mesenchymal stem cells
**ADMSCs**	adipose-derived mesenchymal stem cells
**hAMSCs**	human amniotic mesenchymal stromal cells
**eMSCs**	endometrial mesenchymal stem cells
**EVs**	extracellular vesicles
**MSCs-Exos**	MSC-derived exosomes
**IUDs**	intrauterine devices
**ECM**	extracellular matrix
**TNF-α**	tumor necrosis factor alpha
**IL**	interleukin
**IL-1α**	interleukin-1α
**VEGF**	vascular endothelial-derived growth factor
**D&C**	blind dilation and curettage
**AFS**	American Fertility Society
**PRP**	Platelet-rich plasma
**ECs**	endothelial cells
**EPCs**	endothelial progenitor cells
**dECM**	decellularized extracellular matrix
**ESCRT**	endosomal sorting complex required for the transport
**ADMSC-Exos**	exosomes isolated from ADMSCs
**BMSC-Exos**	exosomes isolated from BMSCs
**UC-MSC-Exos**	exosomes isolated from UC-MSCs
**LPS**	lipopolysaccharide
**MMP**	matrix metalloproteinase
**miRNAs**	microRNAs
**ERS**	endoplasmic reticulum stress
**CTF1**	Cardiotropin-1
**HUVECs**	human umbilical vein endothelial cells
**HIF-1α**	hypoxia-inducible factor 1-alpha
**HA-GMA**	methacrylate hyaluronic acid
**HPMA**	N-(2-hydroxypropyl) methylacrylamide
**PEBP**	poly (ethylene glycol)-b-poly (l-phenylalanine)
**PEG**	poly (ethylene glycol)
**ADSC-Exos**	exosomes isolated from ADSCs
**PCL**	poly (Ɛ-caprolactone)
**CSG**	chitosan hydrogel
**MeHA**	methylated hydroxynic acid
**GelMA**	gelatin methacryloyl
**M2-Exos**	M2 macrophage derived exosomes
**QCS**	quaternized chitosan
**OST**	oxidized starch
**SDF-1α**	Stromal Cell-Derived Factor-1α
**HG**	thermo-sensitive hydroxyethyl chitosan/sodium β-glycerophosphate hydrogel
**AD**	alginate-dopamine
**CS**	chondroitin sulfate
**RSF**	regenerated silk fibroin
**PHEMA**	poly hydroxyethyl methacrylate
**EPL**	ε-polylysine
**PGS**	poly glycerol sebacate
**KGF**	keratinocyte growth factor
**G-CSF**	granulate colony-stimulating factor
**FGF**	fibroblast growth factor
**GF**	growth factors
**IGF-1C**	C domain of insulin like growth factor
**SDF-1α**	stromal cell-derived factor-1α
**hiMSC**	human induced pluripotent stem cell-derived mesenchymal stem cell

## References

[B1] Abdollahiyan P. Oroojalian F. Mokhtarzadeh A. de la Guardia M. (2020). Hydrogel-based 3D bioprinting for bone and cartilage tissue engineering. Biotechnol. J. 15 (12), e2000095. 10.1002/biot.202000095 32869529

[B2] Arabpour M. Saghazadeh A. Rezaei N. (2021). Anti-inflammatory and M2 macrophage polarization-promoting effect of mesenchymal stem cell-derived exosomes. Int. Immunopharmacol. 97, 107823. 10.1016/j.intimp.2021.107823 34102486

[B3] Avila-Salas F. Marican A. Pinochet S. Carreno G. Valdes O. Venegas B. (2019). Film dressings based on hydrogels: simultaneous and sustained-release of bioactive compounds with wound healing properties. Pharmaceutics 11 (9), 447. 10.3390/pharmaceutics11090447 31480682 PMC6781310

[B4] Bao M. Feng Q. Zou L. Huang J. Zhu C. Xia W. (2023). Endoplasmic reticulum stress promotes endometrial fibrosis through the TGF-β/SMAD pathway. Reproduction 165 (2), 171–182. 10.1530/REP-22-0294 36342661

[B5] Benor A. Gay S. DeCherney A. (2020). An update on stem cell therapy for Asherman syndrome. J. Assist. Reprod. Genet. 37 (7), 1511–1529. 10.1007/s10815-020-01801-x 32445154 PMC7376809

[B6] Bian D. Wu Y. Song G. Azizi R. Zamani A. (2022). The application of mesenchymal stromal cells (MSCs) and their derivative exosome in skin wound healing: A comprehensive review. Stem Cell Res. Ther. 13 (1), 24. 10.1186/s13287-021-02697-9 35073970 PMC8785459

[B7] Bittner S. M. Pearce H. A. Hogan K. J. Smoak M. M. Guo J. L. Melchiorri A. J. (2021). Swelling behaviors of 3D printed hydrogel and hydrogel-microcarrier composite scaffolds. Tissue Eng. Part A 27 (11-12), 665–678. 10.1089/ten.TEA.2020.0377 33470161 PMC8349726

[B8] Boniakowski A. E. Kimball A. S. Jacobs B. N. Kunkel S. L. Gallagher K. A. (2017). Macrophage-mediated inflammation in normal and diabetic wound healing. J. Immunol. 199 (1), 17–24. 10.4049/jimmunol.1700223 28630109

[B9] Bos-Mikich A. de Oliveira R. Frantz N. (2018). Platelet-rich plasma therapy and reproductive medicine. J. Assist. Reprod. Genet. 35 (5), 753–756. 10.1007/s10815-018-1159-8 29564738 PMC5984895

[B10] Bosteels J. Kasius J. Weyers S. Broekmans F. J. Mol B. W. D'Hooghe T. M. (2015). Hysteroscopy for treating subfertility associated with suspected major uterine cavity abnormalities. Cochrane Database Syst. Rev. 2, CD009461. 10.1002/14651858.CD009461.pub3 25701429

[B11] Bradley L. D. (2002). Complications in hysteroscopy: prevention, treatment and legal risk. Curr. Opin. Obstet. Gynecol. 14 (4), 409–415. 10.1097/00001703-200208000-00008 12151831

[B12] Cai Y. Wu F. Yu Y. Liu Y. Shao C. Gu H. (2019). Porous scaffolds from droplet microfluidics for prevention of intrauterine adhesion. Acta Biomater. 84, 222–230. 10.1016/j.actbio.2018.11.016 30476581

[B13] Cao H. Duan L. Zhang Y. Cao J. Zhang K. (2021). Current hydrogel advances in physicochemical and biological response-driven biomedical application diversity. Signal Transduct. Target Ther. 6 (1), 426. 10.1038/s41392-021-00830-x 34916490 PMC8674418

[B14] Cao J. Liu D. Zhao S. Yuan L. Huang Y. Ma J. (2020). Estrogen attenuates TGF-β1-induced EMT in intrauterine adhesion by activating Wnt/β-catenin signaling pathway. Braz J. Med. Biol. Res. 53 (8), e9794. 10.1590/1414-431x20209794 32638833 PMC7346761

[B15] Cao Y. Sun H. Zhu H. Zhu X. Tang X. Yan G. (2018). Allogeneic cell therapy using umbilical cord MSCs on collagen scaffolds for patients with recurrent uterine adhesion: a phase I clinical trial. Stem Cell Res. Ther. 9 (1), 192. 10.1186/s13287-018-0904-3 29996892 PMC6042450

[B16] Cen J. Zhang Y. Bai Y. Ma S. Zhang C. Jin L. (2022). Research progress of stem cell therapy for endometrial injury. Mater Today Bio 16, 100389. 10.1016/j.mtbio.2022.100389 PMC9403503 36033375

[B17] Cha G. D. Lee W. H. Sunwoo S. H. Kang D. Kang T. Cho K. W. (2022). Multifunctional injectable hydrogel for *in vivo* diagnostic and therapeutic applications. ACS Nano 16 (1), 554–567. 10.1021/acsnano.1c07649 35014797

[B18] Chen F. Gong Y. Jiang N. Xiao J. Wang Y. Chen L. (2022). Transplantation of bFGF-transfected bone mesenchymal stem cells on collagen scaffolds promotes the regeneration of injured rat endometrium. Am. J. Transl. Res. 14 (9), 6712–6725. 36247308 PMC9556468

[B19] Chen J. M. Huang Q. Y. Zhao Y. X. Chen W. H. Lin S. Shi Q. Y. (2021). The latest developments in immunomodulation of mesenchymal stem cells in the treatment of intrauterine adhesions, both allogeneic and autologous. Front. Immunol. 12, 785717. 10.3389/fimmu.2021.785717 34868069 PMC8634714

[B20] Chen L. Li L. Mo Q. Zhang X. Chen C. Wu Y. (2023a). An injectable gelatin/sericin hydrogel loaded with human umbilical cord mesenchymal stem cells for the treatment of uterine injury. Bioeng. Transl. Med. 8 (1), e10328. 10.1002/btm2.10328 36684066 PMC9842051

[B21] Chen L. Tredget E. E. Wu P. Y. Wu Y. (2008). Paracrine factors of mesenchymal stem cells recruit macrophages and endothelial lineage cells and enhance wound healing. PLoS One 3 (4), e1886. 10.1371/journal.pone.0001886 18382669 PMC2270908

[B22] Chen L. Yu C. Xiong Y. Chen K. Liu P. Panayi A. C. (2023b). Multifunctional hydrogel enhances bone regeneration through sustained release of Stromal Cell-Derived Factor-1α and exosomes. Bioact. Mater 25, 460–471. 10.1016/j.bioactmat.2022.07.030 37056272 PMC10087917

[B23] Cheng F. M. Chen H. X. Li H. D. (2021). Recent progress on hydrogel actuators. J. Mater Chem. B 9 (7), 1762–1780. 10.1039/d0tb02524k 33527974

[B24] Cheng Y. H. Tsai N. C. Chen Y. J. Weng P. L. Chang Y. C. Cheng J. H. (2022). Extracorporeal shock wave therapy combined with platelet-rich plasma during preventive and therapeutic stages of intrauterine adhesion in a rat model. Biomedicines 10 (2), 476. 10.3390/biomedicines10020476 35203684 PMC8962268

[B25] Chi Y. He P. Lei L. Lan Y. Hu J. Meng Y. (2018). Transdermal estrogen gel and oral aspirin combination therapy improves fertility prognosis via the promotion of endometrial receptivity in moderate to severe intrauterine adhesion. Mol. Med. Rep. 17 (5), 6337–6344. 10.3892/mmr.2018.8685 29512784 PMC5928622

[B26] Cholkeri-Singh A. Sasaki K. J. (2016). Hysteroscopy safety. Curr. Opin. Obstet. Gynecol. 28 (4), 250–254. 10.1097/GCO.0000000000000289 27258237

[B27] Conforti A. Alviggi C. Mollo A. De Placido G. Magos A. (2013). The management of asherman syndrome: A review of literature. Reprod. Biol. Endocrinol. 11, 118. 10.1186/1477-7827-11-118 24373209 PMC3880005

[B28] Crha K. Ventruba P. Zakova J. Jeseta M. Pilka R. Vodicka J. (2019). The role of mesenchymal-epithelial transition in endometrial function and receptivity. Ceska Gynekol. 84 (5), 371–375. 31826635

[B29] Das M. Mayilsamy K. Mohapatra S. S. Mohapatra S. (2019). Mesenchymal stem cell therapy for the treatment of traumatic brain injury: progress and prospects. Rev. Neurosci. 30 (8), 839–855. 10.1515/revneuro-2019-0002 31203262

[B30] Ding L. Li X. Sun H. Su J. Lin N. Peault B. (2014). Transplantation of bone marrow mesenchymal stem cells on collagen scaffolds for the functional regeneration of injured rat uterus. Biomaterials 35 (18), 4888–4900. 10.1016/j.biomaterials.2014.02.046 24680661

[B31] Dong L. Wang S. J. Zhao X. R. Zhu Y. F. Yu J. K. (2017). 3D- printed poly(ε-caprolactone) scaffold integrated with cell-laden chitosan hydrogels for bone tissue engineering. Sci. Rep. 7 (1), 13412. 10.1038/s41598-017-13838-7 29042614 PMC5645328

[B32] Dreisler E. Kjer J. J. (2019). <p&gt;Asherman&amp;rsquo;s syndrome: current perspectives on diagnosis and management&lt;/p&gt;. Int. J. Womens Health 11, 191–198. 10.2147/IJWH.S165474 30936754 PMC6430995

[B33] Fan L. Liu C. Chen X. Zheng L. Zou Y. Wen H. (2022). Exosomes-loaded electroconductive hydrogel synergistically promotes tissue repair after spinal cord injury via immunoregulation and enhancement of myelinated axon growth. Adv. Sci. (Weinh) 9 (13), e2105586. 10.1002/advs.202105586 35253394 PMC9069372

[B34] Feng H. Liu Q. Deng Z. Li H. Zhang H. Song J. (2022). Human umbilical cord mesenchymal stem cells ameliorate erectile dysfunction in rats with diabetes mellitus through the attenuation of ferroptosis. Stem Cell Res. Ther. 13 (1), 450. 10.1186/s13287-022-03147-w 36064453 PMC9444126

[B35] Ferroni L. Gardin C. D'Amora U. Calza L. Ronca A. Tremoli E. (2022). Exosomes of mesenchymal stem cells delivered from methacrylated hyaluronic acid patch improve the regenerative properties of endothelial and dermal cells. Biomater. Adv. 139, 213000. 10.1016/j.bioadv.2022.213000 35891601

[B36] Fu X. Liu G. Halim A. Ju Y. Luo Q. Song A. G. (2019). Mesenchymal stem cell migration and tissue repair. Cells 8 (8), 784. 10.3390/cells8080784 31357692 PMC6721499

[B37] Gao J. Wen J. Hu D. Liu K. Zhang Y. Zhao X. (2022). Bottlebrush inspired injectable hydrogel for rapid prevention of postoperative and recurrent adhesion. Bioact. Mater 16, 27–46. 10.1016/j.bioactmat.2022.02.015 35386330 PMC8958549

[B38] Gharibeh N. Aghebati-Maleki L. Madani J. Pourakbari R. Yousefi M. Ahmadian Heris J. (2022). Cell-based therapy in thin endometrium and Asherman syndrome. Stem Cell Res. Ther. 13 (1), 33. 10.1186/s13287-021-02698-8 35090547 PMC8796444

[B39] Goncalves N. J. N. Frantz N. de Oliveira R. M. (2020). Platelet-rich plasma (PRP) therapy: an approach in reproductive medicine based on successful animal models. Anim. Reprod. 16 (1), 93–98. 10.21451/1984-3143-ar2018-0093 33299482 PMC7720930

[B40] Grant C. M. Kyprianou N. (2013). Epithelial mesenchymal transition (EMT) in prostate growth and tumor progression. Transl. Androl. Urol. 2 (3), 202–211. 10.3978/j.issn.2223-4683.2013.09.04 25346895 PMC4208065

[B41] Guan M. Liu C. Zheng Q. Chu G. Wang H. Jin J. (2023). Exosome-laden injectable self-healing hydrogel based on quaternized chitosan and oxidized starch attenuates disc degeneration by suppressing nucleus pulposus senescence. Int. J. Biol. Macromol. 232, 123479. 10.1016/j.ijbiomac.2023.123479 36731695

[B42] Guan P. Cui R. Wang Q. Sun Y. (2022). A 3D hydrogel loaded with exosomes derived from bone marrow stem cells promotes cartilage repair in rats by modulating immunological microenvironment. Nan Fang. Yi Ke Da Xue Xue Bao 42 (4), 528–537. 10.12122/j.issn.1673-4254.2022.04.08 35527488 PMC9085588

[B43] Guo Y. Shi X. Song D. Liu Y. Huang X. Xiao Y. (2022). The efficacy of auto-cross-linked hyaluronic acid gel in addition to oestradiol and intrauterine balloon insertion in the prevention of adhesion reformation after hysteroscopic adhesiolysis. Reprod. Biomed. Online 45 (3), 501–507. 10.1016/j.rbmo.2022.04.017 35760666

[B44] Han C. Y. Rho H. S. Kim A. Kim T. H. Jang K. Jun D. W. (2018). FXR inhibits endoplasmic reticulum stress-induced NLRP3 inflammasome in hepatocytes and ameliorates liver injury. Cell Rep. 24 (11), 2985–2999. 10.1016/j.celrep.2018.07.068 30208322

[B45] Han M. Yang H. Lu X. Li Y. Liu Z. Li F. (2022). Three-Dimensional-cultured MSC-derived exosome-hydrogel hybrid microneedle array patch for spinal cord repair. Nano Lett. 22 (15), 6391–6401. 10.1021/acs.nanolett.2c02259 35876503

[B46] Hapangama D. K. Kamal A. M. Bulmer J. N. (2015). Estrogen receptor β: the guardian of the endometrium. Hum. Reprod. Update 21 (2), 174–193. 10.1093/humupd/dmu053 25305176

[B47] He C. Zheng S. Luo Y. Wang B. (2018). Exosome theranostics: biology and translational medicine. Theranostics 8 (1), 237–255. 10.7150/thno.21945 29290805 PMC5743472

[B48] He X. Dong Z. Cao Y. Wang H. Liu S. Liao L. (2019). MSC-derived exosome promotes M2 polarization and enhances cutaneous wound healing. Stem Cells Int. 2019, 1–16. 10.1155/2019/7132708 PMC6754952 31582986

[B49] Heidarian P. Gharaie S. Yousefi H. Paulino M. Kaynak A. Varley R. (2022). A 3D printable dynamic nanocellulose/nanochitin self-healing hydrogel and soft strain sensor. Carbohydr. Polym. 291, 119545. 10.1016/j.carbpol.2022.119545 35698375

[B50] Hoang D. H. Nguyen T. D. Nguyen H. P. Nguyen X. H. Do P. T. X. Dang V. D. (2020). Differential wound healing capacity of mesenchymal stem cell-derived exosomes originated from bone marrow, adipose tissue and umbilical cord under serum- and xeno-free condition. Front. Mol. Biosci. 7, 119. 10.3389/fmolb.2020.00119 32671095 PMC7327117

[B51] Hong H. Seo Y. B. Kim D. Y. Lee J. S. Lee Y. J. Lee H. (2020). Digital light processing 3D printed silk fibroin hydrogel for cartilage tissue engineering. Biomaterials 232, 119679. 10.1016/j.biomaterials.2019.119679 31865191

[B52] Hooker A. B. de Leeuw R. A. Twisk J. W. R. Brolmann H. A. M. Huirne J. A. F. (2021). Reproductive performance of women with and without intrauterine adhesions following recurrent dilatation and curettage for miscarriage: long-term follow-up of a randomized controlled trial. Hum. Reprod. 36 (1), 70–81. 10.1093/humrep/deaa289 33320197 PMC7801791

[B53] Hooker A. B. Lemmers M. Thurkow A. L. Heymans M. W. Opmeer B. C. Brolmann H. A. (2014). Systematic review and meta-analysis of intrauterine adhesions after miscarriage: prevalence, risk factors and long-term reproductive outcome. Hum. Reprod. Update 20 (2), 262–278. 10.1093/humupd/dmt045 24082042

[B54] Hossain Rakin R. Kumar H. Rajeev A. Natale G. Menard F. Li I. T. S. (2021). Tunable metacrylated hyaluronic acid-based hybrid bioinks for stereolithography 3D bioprinting. Biofabrication 13 (4), 044109. 10.1088/1758-5090/ac25cb 34507314

[B55] Hu J. Zhang L. Liechty C. Zgheib C. Hodges M. M. Liechty K. W. (2020a). Long noncoding RNA GAS5 regulates macrophage polarization and diabetic wound healing. J. Invest. Dermatol 140 (8), 1629–1638. 10.1016/j.jid.2019.12.030 32004569 PMC7384923

[B56] Hu M. S. Borrelli M. R. Lorenz H. P. Longaker M. T. Wan D. C. (2018). Mesenchymal stromal cells and cutaneous wound healing: A comprehensive review of the background, role, and therapeutic potential. Stem Cells Int. 2018, 1–13. 10.1155/2018/6901983 PMC5985130 29887893

[B57] Hu M. Zhang Y. Li X. Cui P. Li J. Brannstrom M. (2020b). Alterations of endometrial epithelial-mesenchymal transition and MAPK signalling components in women with PCOS are partially modulated by metformin *in vitro* . Mol. Hum. Reprod. 26 (5), 312–326. 10.1093/molehr/gaaa023 32202622

[B58] Hu Q. Xie N. Liao K. Huang J. Yang Q. Zhou Y. (2022). An injectable thermosensitive Pluronic F127/hyaluronic acid hydrogel loaded with human umbilical cord mesenchymal stem cells and asiaticoside microspheres for uterine scar repair. Int. J. Biol. Macromol. 219, 96–108. 10.1016/j.ijbiomac.2022.07.161 35902020

[B59] Huang X. W. Lin M. M. Zhao H. Q. Powell M. Wang Y. Q. Zheng R. R. (2020a). A prospective randomized controlled trial comparing two different treatments of intrauterine adhesions. Reprod. Biomed. Online 40 (6), 835–841. 10.1016/j.rbmo.2020.02.013 32376313

[B60] Huang Y. He B. Wang L. Yuan B. Shu H. Zhang F. (2020b). Bone marrow mesenchymal stem cell-derived exosomes promote rotator cuff tendon-bone healing by promoting angiogenesis and regulating M1 macrophages in rats. Stem Cell Res. Ther. 11 (1), 496. 10.1186/s13287-020-02005-x 33239091 PMC7687785

[B61] Isaac R. Reis F. C. G. Ying W. Olefsky J. M. (2021). Exosomes as mediators of intercellular crosstalk in metabolism. Cell Metab. 33 (9), 1744–1762. 10.1016/j.cmet.2021.08.006 34496230 PMC8428804

[B62] Jang H. Y. Myoung S. M. Choe J. M. Kim T. Cheon Y. P. Kim Y. M. (2017). Effects of autologous platelet-rich plasma on regeneration of damaged endometrium in female rats. Yonsei Med. J. 58 (6), 1195–1203. 10.3349/ymj.2017.58.6.1195 29047244 PMC5653485

[B63] Ji W. Hou B. Lin W. Wang L. Zheng W. Li W. (2020a). 3D Bioprinting a human iPSC-derived MSC-loaded scaffold for repair of the uterine endometrium. Acta Biomater. 116, 268–284. 10.1016/j.actbio.2020.09.012 32911103

[B64] Ji X. Yuan X. Ma L. Bi B. Zhu H. Lei Z. (2020b). Mesenchymal stem cell-loaded thermosensitive hydroxypropyl chitin hydrogel combined with a three-dimensional-printed poly(ε-caprolactone)/nano-hydroxyapatite scaffold to repair bone defects via osteogenesis, angiogenesis and immunomodulation. Theranostics 10 (2), 725–740. 10.7150/thno.39167 31903147 PMC6929983

[B65] Jiang P. Tang X. Wang H. Dai C. Su J. Zhu H. (2019). Collagen-binding basic fibroblast growth factor improves functional remodeling of scarred endometrium in uterine infertile women: A pilot study. Sci. China Life Sci. 62 (12), 1617–1629. 10.1007/s11427-018-9520-2 31515729

[B66] Jiang T. Liu S. Wu Z. Li Q. Ren S. Chen J. (2022). ADSC-exo@MMP-PEG smart hydrogel promotes diabetic wound healing by optimizing cellular functions and relieving oxidative stress. Mater Today Bio 16, 100365. 10.1016/j.mtbio.2022.100365 PMC9364034 35967739

[B67] Jin P. Liu L. Chen X. Cheng L. Zhang W. Zhong G. (2022). Applications and prospects of different functional hydrogels in meniscus repair. Front. Bioeng. Biotechnol. 10, 1082499. 10.3389/fbioe.2022.1082499 36568293 PMC9773848

[B68] Johary J. Xue M. Zhu X. Xu D. Velu P. P. (2014). Efficacy of estrogen therapy in patients with intrauterine adhesions: systematic review. J. Minim. Invasive Gynecol. 21 (1), 44–54. 10.1016/j.jmig.2013.07.018 23933351

[B69] Ju Y. Hu Y. Yang P. Xie X. Fang B. (2023). Extracellular vesicle-loaded hydrogels for tissue repair and regeneration. Mater Today Bio 18, 100522. 10.1016/j.mtbio.2022.100522 PMC9803958 36593913

[B70] Kalluri R. LeBleu V. S. (2020). The biology, function, and biomedical applications of exosomes. Science 367 (6478), eaau6977. 10.1126/science.aau6977 32029601 PMC7717626

[B71] Kanlaya R. Kapincharanon C. Fong-Ngern K. Thongboonkerd V. (2022). Induction of mesenchymal-epithelial transition (met) by epigallocatechin-3-gallate to reverse epithelial-mesenchymal transition (EMT) in SNAI1-overexpressed renal cells: A potential anti-fibrotic strategy. J. Nutr. Biochem. 107, 109066. 10.1016/j.jnutbio.2022.109066 35609852

[B72] Khan Z. Goldberg J. M. (2018). Hysteroscopic management of asherman's syndrome. J. Minim. Invasive Gynecol. 25 (2), 218–228. 10.1016/j.jmig.2017.09.020 29024798

[B73] Khayambashi P. Iyer J. Pillai S. Upadhyay A. Zhang Y. Tran S. D. (2021). Hydrogel encapsulation of mesenchymal stem cells and their derived exosomes for tissue engineering. Int. J. Mol. Sci. 22 (2), 684. 10.3390/ijms22020684 33445616 PMC7827932

[B74] Kim Y. Y. Park K. H. Kim Y. J. Kim M. S. Liu H. C. Rosenwaks Z. (2019). Synergistic regenerative effects of functionalized endometrial stromal cells with hyaluronic acid hydrogel in a murine model of uterine damage. Acta Biomater. 89, 139–151. 10.1016/j.actbio.2019.03.032 30898731

[B75] Kou L. Jiang X. Xiao S. Zhao Y.-Z. Yao Q. Chen R. (2020a). Therapeutic options and drug delivery strategies for the prevention of intrauterine adhesions. J. Control. Release Official J. Control. Release Soc. 318, 25–37. 10.1016/j.jconrel.2019.12.007 31830539

[B76] Kou L. Jiang X. Xiao S. Zhao Y. Z. Yao Q. Chen R. (2020b). Therapeutic options and drug delivery strategies for the prevention of intrauterine adhesions. J. Control Release 318, 25–37. 10.1016/j.jconrel.2019.12.007 31830539

[B77] Kou M. Huang L. Yang J. Chiang Z. Chen S. Liu J. (2022). Mesenchymal stem cell-derived extracellular vesicles for immunomodulation and regeneration: A next generation therapeutic tool? Cell Death Dis. 13 (7), 580. 10.1038/s41419-022-05034-x 35787632 PMC9252569

[B78] Kwak G. Cheng J. Kim H. Song S. Lee S. J. Yang Y. (2022). Sustained exosome-guided macrophage polarization using hydrolytically degradable PEG hydrogels for cutaneous wound healing: identification of key proteins and MiRNAs, and sustained release formulation. Small 18 (15), e2200060. 10.1002/smll.202200060 35229462

[B79] Li B. Cao Y. Sun M. Feng H. (2021a). Expression, regulation, and function of exosome-derived miRNAs in cancer progression and therapy. FASEB J. 35 (10), e21916. 10.1096/fj.202100294RR 34510546

[B80] Li C. Zheng Z. Jia J. Zhang W. Qin L. Zhang W. (2022a). Preparation and characterization of photocurable composite extracellular matrix-methacrylated hyaluronic acid bioink. J. Mater Chem. B 10 (22), 4242–4253. 10.1039/d2tb00548d 35579559

[B81] Li Q. Cui J. Huang H. Yue Z. Chang Y. Li N. (2020a). IGF-1C domain-modified chitosan hydrogel accelerates cutaneous wound healing by promoting angiogenesis. Future Med. Chem. 12 (13), 1239–1251. 10.4155/fmc-2020-0071 32351127

[B82] Li R. Li D. Wang H. Chen K. Wang S. Xu J. (2022b). Exosomes from adipose-derived stem cells regulate M1/M2 macrophage phenotypic polarization to promote bone healing via miR-451a/MIF. Stem Cell Res. Ther. 13 (1), 149. 10.1186/s13287-022-02823-1 35395782 PMC8994256

[B83] Li X. Lv H. F. Zhao R. Ying M. F. Samuriwo A. T. Zhao Y. Z. (2021b). Recent developments in bio-scaffold materials as delivery strategies for therapeutics for endometrium regeneration. Mater Today Bio 11, 100101. 10.1016/j.mtbio.2021.100101 PMC8138682 34036261

[B84] Li X. T. Zhao J. Xu D. S. Zhang Y. Zhou S. T. (2020c). Bone marrow mesenchymal stem cell exosomes promote brain microvascular endothelial cell proliferation and migration in rats. Sichuan Da Xue Xue Bao Yi Xue Ban. 51 (5), 599–604. 10.12182/20200960207 32975071

[B85] Li X. Zhang Y. Wang Y. Zhao D. Sun C. Zhou S. (2020b). Exosomes derived from CXCR4-overexpressing BMSC promoted activation of microvascular endothelial cells in cerebral ischemia/reperfusion injury. Neural Plast. 2020, 1–13. 10.1155/2020/8814239 PMC7762674 33381162

[B86] Liang Y. Duan L. Lu J. Xia J. (2021). Engineering exosomes for targeted drug delivery. Theranostics 11 (7), 3183–3195. 10.7150/thno.52570 33537081 PMC7847680

[B87] Lin J. Wang Z. Huang J. Tang S. Saiding Q. Zhu Q. (2021). Microenvironment-protected exosome-hydrogel for facilitating endometrial regeneration, fertility restoration, and live birth of offspring. Small 17 (11), e2007235. 10.1002/smll.202007235 33590681

[B88] Lin Y. Dong S. Ye X. Liu J. Li J. Zhang Y. (2022). Synergistic regenerative therapy of thin endometrium by human placenta-derived mesenchymal stem cells encapsulated within hyaluronic acid hydrogels. Stem Cell Res. Ther. 13 (1), 66. 10.1186/s13287-022-02717-2 35135594 PMC8822809

[B89] Liu F. Hu S. Yang H. Li Z. Huang K. Su T. (2019). Hyaluronic acid hydrogel integrated with mesenchymal stem cell-secretome to treat endometrial injury in a rat model of asherman's syndrome. Adv. Healthc. Mater 8 (14), e1900411. 10.1002/adhm.201900411 31148407 PMC7045702

[B90] Liu J. Qiu R. Liu R. Song P. Lin P. Chen H. (2022a). Autophagy mediates Escherichia coli-induced cellular inflammatory injury by regulating calcium mobilization, mitochondrial dysfunction, and endoplasmic reticulum stress. Int. J. Mol. Sci. 23 (22), 14174. 10.3390/ijms232214174 36430657 PMC9698444

[B91] Liu S. Li R. Dou K. Li K. Zhou Q. Fu Q. (2023a). Injectable thermo-sensitive hydrogel containing ADSC-derived exosomes for the treatment of cavernous nerve injury. Carbohydr. Polym. 300, 120226. 10.1016/j.carbpol.2022.120226 36372471

[B92] Liu W. Li L. Rong Y. Qian D. Chen J. Zhou Z. (2020a). Hypoxic mesenchymal stem cell-derived exosomes promote bone fracture healing by the transfer of miR-126. Acta Biomater. 103, 196–212. 10.1016/j.actbio.2019.12.020 31857259

[B93] Liu W. Rong Y. Wang J. Zhou Z. Ge X. Ji C. (2020b). Exosome-shuttled miR-216a-5p from hypoxic preconditioned mesenchymal stem cells repair traumatic spinal cord injury by shifting microglial M1/M2 polarization. J. Neuroinflammation 17 (1), 47. 10.1186/s12974-020-1726-7 32019561 PMC7001326

[B94] Liu X. Wu C. Zhang Y. Chen S. Ding J. Chen Z. (2023b). Hyaluronan-based hydrogel integrating exosomes for traumatic brain injury repair by promoting angiogenesis and neurogenesis. Carbohydr. Polym. 306, 120578. 10.1016/j.carbpol.2023.120578 36746568

[B95] Liu Y. Cai J. Luo X. Wen H. Luo Y. (2020c). Collagen scaffold with human umbilical cord mesenchymal stem cells remarkably improves intrauterine adhesions in a rat model. Gynecol. Obstet. Invest. 85 (3), 267–276. 10.1159/000505691 32289792

[B96] Liu Y. R. Liu B. Yang B. P. Lan Y. Chi Y. G. (2022b). Efficacy of hyaluronic acid on the prevention of intrauterine adhesion and the improvement of fertility: A meta-analysis of randomized trials. Complement. Ther. Clin. Pract. 47, 101575. 10.1016/j.ctcp.2022.101575 35349823

[B97] Liu Y. Wong C. W. Chang S. W. Hsu S. H. (2021). An injectable, self-healing phenol-functionalized chitosan hydrogel with fast gelling property and visible light-crosslinking capability for 3D printing. Acta Biomater. 122, 211–219. 10.1016/j.actbio.2020.12.051 33444794

[B98] Long S. Colson L. (2021). Intrauterine device insertion and removal. Prim. Care 48 (4), 531–544. 10.1016/j.pop.2021.07.001 34752267

[B99] Lopez-Martinez S. Rodriguez-Eguren A. de Miguel-Gomez L. Frances-Herrero E. Faus A. Diaz A. (2021). Bioengineered endometrial hydrogels with growth factors promote tissue regeneration and restore fertility in murine models. Acta Biomater. 135, 113–125. 10.1016/j.actbio.2021.08.025 34428563

[B100] Lu G. D. Cheng P. Liu T. Wang Z. (2020). BMSC-derived exosomal miR-29a promotes angiogenesis and osteogenesis. Front. Cell Dev. Biol. 8, 608521. 10.3389/fcell.2020.608521 33363169 PMC7755650

[B101] Lv H. Liu H. Sun T. Wang H. Zhang X. Xu W. (2022). Exosome derived from stem cell: A promising therapeutics for wound healing. Front. Pharmacol. 13, 957771. 10.3389/fphar.2022.957771 36003496 PMC9395204

[B102] Lv Q. Wang L. Luo X. Chen X. (2021). Adult stem cells in endometrial regeneration: molecular insights and clinical applications. Mol. Reprod. Dev. 88 (6), 379–394. 10.1002/mrd.23476 34014590 PMC8362170

[B103] Ma J. Zhan H. Li W. Zhang L. Yun F. Wu R. (2021). Recent trends in therapeutic strategies for repairing endometrial tissue in intrauterine adhesion. Biomater. Res. 25 (1), 40. 10.1186/s40824-021-00242-6 34819167 PMC8611984

[B104] Malhotra S. Hu M. S. Marshall C. D. Leavitt T. Cheung A. T. Gonzalez J. G. (2016). Mesenchymal stromal cells as cell-based therapeutics for wound healing. Stem Cells Int. 2016, 1–6. 10.1155/2016/4157934 PMC4757746 26966438

[B105] Mandrycky C. Wang Z. Kim K. Kim D. H. (2016). 3D bioprinting for engineering complex tissues. Biotechnol. Adv. 34 (4), 422–434. 10.1016/j.biotechadv.2015.12.011 26724184 PMC4879088

[B106] Mao C. Xiang Y. Liu X. Cui Z. Yang X. Yeung K. W. K. (2017). Photo-Inspired antibacterial activity and wound healing acceleration by hydrogel embedded with Ag/Ag@AgCl/ZnO nanostructures. ACS Nano 11 (9), 9010–9021. 10.1021/acsnano.7b03513 28825807

[B107] Marconi G. D. Fonticoli L. Rajan T. S. Pierdomenico S. D. Trubiani O. Pizzicannella J. (2021). Epithelial-mesenchymal transition (EMT): the type-2 EMT in wound healing, tissue regeneration and organ fibrosis. Cells 10 (7), 1587. 10.3390/cells10071587 34201858 PMC8307661

[B108] Marofi F. Alexandrovna K. I. Margiana R. Bahramali M. Suksatan W. Abdelbasset W. K. (2021). MSCs and their exosomes: A rapidly evolving approach in the context of cutaneous wounds therapy. Stem Cell Res. Ther. 12 (1), 597. 10.1186/s13287-021-02662-6 34863308 PMC8642895

[B109] Martinez-Martinez E. Brugnolaro C. Ibarrola J. Ravassa S. Buonafine M. Lopez B. (2019). CT-1 (Cardiotrophin-1)-Gal-3 (Galectin-3) Axis in cardiac fibrosis and inflammation. Hypertension 73 (3), 602–611. 10.1161/HYPERTENSIONAHA.118.11874 30612490

[B110] McGettrick A. F. O'Neill L. A. J. (2020). The role of HIF in immunity and inflammation. Cell Metab. 32 (4), 524–536. 10.1016/j.cmet.2020.08.002 32853548

[B111] Mi B. Chen L. Xiong Y. Yang Y. Panayi A. C. Xue H. (2022). Osteoblast/osteoclast and immune cocktail therapy of an exosome/drug delivery multifunctional hydrogel accelerates fracture repair. ACS Nano 16 (1), 771–782. 10.1021/acsnano.1c08284 34979087

[B112] Mori M. A. Ludwig R. G. Garcia-Martin R. Brandao B. B. Kahn C. R. (2019). Extracellular miRNAs: from biomarkers to mediators of physiology and disease. Cell Metab. 30 (4), 656–673. 10.1016/j.cmet.2019.07.011 31447320 PMC6774861

[B113] Mu J. Li L. Wu J. Huang T. Zhang Y. Cao J. (2022). Hypoxia-stimulated mesenchymal stem cell-derived exosomes loaded by adhesive hydrogel for effective angiogenic treatment of spinal cord injury. Biomater. Sci. 10 (7), 1803–1811. 10.1039/d1bm01722e 35234220

[B114] Nakao Y. Fukuda T. Zhang Q. Sanui T. Shinjo T. Kou X. (2021). Exosomes from TNF-alpha-treated human gingiva-derived MSCs enhance M2 macrophage polarization and inhibit periodontal bone loss. Acta Biomater. 122, 306–324. 10.1016/j.actbio.2020.12.046 33359765 PMC7897289

[B115] Nie N. Gong L. Jiang D. Liu Y. Zhang J. Xu J. (2023). 3D bio-printed endometrial construct restores the full-thickness morphology and fertility of injured uterine endometrium. Acta Biomater. 157, 187–199. 10.1016/j.actbio.2022.12.016 36521675

[B116] Owusu-Akyaw A. Krishnamoorthy K. Goldsmith L. T. Morelli S. S. (2019). The role of mesenchymal-epithelial transition in endometrial function. Hum. Reprod. Update 25 (1), 114–133. 10.1093/humupd/dmy035 30407544

[B117] Padhi A. Nain A. S. (2020). ECM in differentiation: A review of matrix structure, composition and mechanical properties. Ann. Biomed. Eng. 48 (3), 1071–1089. 10.1007/s10439-019-02337-7 31485876

[B118] Pan Y. Zhao Y. Kuang R. Liu H. Sun D. Mao T. (2020). Injectable hydrogel-loaded nano-hydroxyapatite that improves bone regeneration and alveolar ridge promotion. Mater Sci. Eng. C Mater Biol. Appl. 116, 111158. 10.1016/j.msec.2020.111158 32806272

[B119] Puente Gonzalo E. Alonso Pacheco L. Vega Jimenez A. Vitale S. G. Raffone A. Lagana A. S. (2021). Intrauterine infusion of platelet-rich plasma for severe asherman syndrome: A cutting-edge approach. Updat. Surg. 73 (6), 2355–2362. 10.1007/s13304-020-00828-0 32514742

[B120] Rungsiwiwut R. Virutamasen P. Pruksananonda K. (2021). Mesenchymal stem cells for restoring endometrial function: an infertility perspective. Reprod. Med. Biol. 20 (1), 13–19. 10.1002/rmb2.12339 33488279 PMC7812475

[B121] Salazar C. A. Isaacson K. Morris S. (2017). A comprehensive review of asherman's syndrome: causes, symptoms and treatment options. Curr. Opin. Obstet. Gynecol. 29 (4), 249–256. 10.1097/GCO.0000000000000378 28582327

[B122] Salma U. Xue M. Md Sayed A. S. Xu D. (2014). Efficacy of intrauterine device in the treatment of intrauterine adhesions. Biomed. Res. Int. 2014, 1–15. 10.1155/2014/589296 PMC4165200 25254212

[B123] Salunkhe S. DheerajBasak M. Chitkara D. Mittal A. (2020). Surface functionalization of exosomes for target-specific delivery and *in vivo* imaging & tracking: strategies and significance. J. Control Release 326, 599–614. 10.1016/j.jconrel.2020.07.042 32730952

[B124] Scully D. Naseem K. M. Matsakas A. (2018). Platelet biology in regenerative medicine of skeletal muscle. Acta Physiol. (Oxf) 223 (3), e13071. 10.1111/apha.13071 29633517

[B125] Sharma S. Tiwari S. (2020). RETRACTED: A review on biomacromolecular hydrogel classification and its applications. Int. J. Biol. Macromol. 162, 737–747. 10.1016/j.ijbiomac.2020.06.110 32553961

[B126] Shen K. Duan A. Cheng J. Yuan T. Zhou J. Song H. (2022). Exosomes derived from hypoxia preconditioned mesenchymal stem cells laden in a silk hydrogel promote cartilage regeneration via the miR-205-5p/PTEN/AKT pathway. Acta Biomater. 143, 173–188. 10.1016/j.actbio.2022.02.026 35202856

[B127] Shi Y. Kang X. Wang Y. Bian X. He G. Zhou M. (2020). Exosomes derived from bone marrow stromal cells (BMSCs) enhance tendon-bone healing by regulating macrophage polarization. Med. Sci. Monit. 26, e923328. 10.12659/MSM.923328 32369458 PMC7218969

[B128] Song Y. T. Liu P. C. Tan J. Zou C. Y. Li Q. J. Li-Ling J. (2021). Stem cell-based therapy for ameliorating intrauterine adhesion and endometrium injury. Stem Cell Res. Ther. 12 (1), 556. 10.1186/s13287-021-02620-2 34717746 PMC8557001

[B129] Sun J. Shen H. Shao L. Teng X. Chen Y. Liu X. (2020). HIF-1α overexpression in mesenchymal stem cell-derived exosomes mediates cardioprotection in myocardial infarction by enhanced angiogenesis. Stem Cell Res. Ther. 11 (1), 373. 10.1186/s13287-020-01881-7 32859268 PMC7455909

[B130] Tamura R. Uemoto S. Tabata Y. (2017). Augmented liver targeting of exosomes by surface modification with cationized pullulan. Acta Biomater. 57, 274–284. 10.1016/j.actbio.2017.05.013 28483695

[B131] Ti D. Hao H. Tong C. Liu J. Dong L. Zheng J. (2015). LPS-preconditioned mesenchymal stromal cells modify macrophage polarization for resolution of chronic inflammation via exosome-shuttled let-7b. J. Transl. Med. 13, 308. 10.1186/s12967-015-0642-6 26386558 PMC4575470

[B132] Wang B. Feng C. Dang J. Zhu Y. Yang X. Zhang T. (2021a). Preparation of fibroblast suppressive poly(ethylene glycol)-b-poly(l-phenylalanine)/Poly(ethylene glycol) hydrogel and its application in intrauterine fibrosis prevention. ACS Biomater. Sci. Eng. 7 (1), 311–321. 10.1021/acsbiomaterials.0c01390 33455202

[B133] Wang C. Wang M. Xu T. Zhang X. Lin C. Gao W. (2019). Engineering bioactive self-healing antibacterial exosomes hydrogel for promoting chronic diabetic wound healing and complete skin regeneration. Theranostics 9 (1), 65–76. 10.7150/thno.29766 30662554 PMC6332800

[B134] Wang H. Liu S. Zhang W. Liu M. Deng C. (2022a). Human umbilical cord mesenchymal stem cell-derived exosome repairs endometrial epithelial cells injury induced by hypoxia via regulating miR-663a/cdkn2a Axis. Oxid. Med. Cell Longev. 2022, 1–15. 10.1155/2022/3082969 PMC9581691 36275892

[B135] Wang J. Yang C. Xie Y. Chen X. Jiang T. Tian J. (2021b). Application of bioactive hydrogels for functional treatment of intrauterine adhesion. Front. Bioeng. Biotechnol. 9, 760943. 10.3389/fbioe.2021.760943 34621732 PMC8490821

[B136] Wang J. Zhu M. Hu Y. Chen R. Hao Z. Wang Y. (2022b). Exosome-hydrogel system in bone tissue engineering: A promising therapeutic strategy. Macromol. Biosci. 23, e2200496. 10.1002/mabi.202200496 36573715

[B137] Wang L. Wang J. Zhou X. Sun J. Zhu B. Duan C. (2020). A new self-healing hydrogel containing hucMSC-derived exosomes promotes bone regeneration. Front. Bioeng. Biotechnol. 8, 564731. 10.3389/fbioe.2020.564731 33042966 PMC7521201

[B138] Wang L. Zhang D. Ren Y. Guo S. Li J. Ma S. (2022c). Injectable hyaluronic acid hydrogel loaded with BMSC and NGF for traumatic brain injury treatment. Mater Today Bio 13, 100201. 10.1016/j.mtbio.2021.100201 PMC8733324 35024600

[B139] Wang X. Wang Q. (2021). Enzyme-Laden bioactive hydrogel for biocatalytic monitoring and regulation. Acc. Chem. Res. 54 (5), 1274–1287. 10.1021/acs.accounts.0c00832 33570397

[B140] Warembourg S. Huberlant S. Garric X. Leprince S. de Tayrac R. Letouzey V. (2015). Prevention and treatment of intra-uterine synechiae: review of the literature. J. Gynecol. Obstet. Biol. Reprod. Paris. 44 (4), 366–379. 10.1016/j.jgyn.2014.10.014 25479692

[B141] Wei C. Pan Y. Zhang Y. Dai Y. Jiang L. Shi L. (2020). Overactivated sonic hedgehog signaling aggravates intrauterine adhesion via inhibiting autophagy in endometrial stromal cells. Cell Death Dis. 11 (9), 755. 10.1038/s41419-020-02956-2 32934215 PMC7492405

[B142] Weimar C. H. Macklon N. S. Post Uiterweer E. D. Brosens J. J. Gellersen B. (2013). The motile and invasive capacity of human endometrial stromal cells: implications for normal and impaired reproductive function. Hum. Reprod. Update 19 (5), 542–557. 10.1093/humupd/dmt025 23827985

[B143] Wen J. Hou B. Lin W. Guo F. Cheng M. Zheng J. (2022). 3D-printed hydrogel scaffold-loaded granulocyte colony-stimulating factor sustained-release microspheres and their effect on endometrial regeneration. Biomater. Sci. 10 (12), 3346–3358. 10.1039/d2bm00109h 35588302

[B144] Wenbo Q. Lijian X. Shuangdan Z. Jiahua Z. Yanpeng T. Xuejun Q. (2020). Controlled releasing of SDF-1α in chitosan-heparin hydrogel for endometrium injury healing in rat model. Int. J. Biol. Macromol. 143, 163–172. 10.1016/j.ijbiomac.2019.11.184 31765745

[B145] Wu P. Zhang B. Shi H. Qian H. Xu W. (2018). MSC-exosome: A novel cell-free therapy for cutaneous regeneration. Cytotherapy 20 (3), 291–301. 10.1016/j.jcyt.2017.11.002 29434006

[B146] Wu Y. Chen L. Scott P. G. Tredget E. E. (2007). Mesenchymal stem cells enhance wound healing through differentiation and angiogenesis. Stem Cells 25 (10), 2648–2659. 10.1634/stemcells.2007-0226 17615264

[B147] Xiao B. Yang W. Lei D. Huang J. Yin Y. Zhu Y. (2019). PGS scaffolds promote the *in vivo* survival and directional differentiation of bone marrow mesenchymal stem cells restoring the morphology and function of wounded rat uterus. Adv. Healthc. Mater 8 (5), e1801455. 10.1002/adhm.201801455 30734535

[B148] Xiao Y. Gu Y. Qin L. Chen L. Chen X. Cui W. (2021). Injectable thermosensitive hydrogel-based drug delivery system for local cancer therapy. Colloids Surf. B Biointerfaces 200, 111581. 10.1016/j.colsurfb.2021.111581 33524696

[B149] Xin L. Lin X. Pan Y. Zheng X. Shi L. Zhang Y. (2019). A collagen scaffold loaded with human umbilical cord-derived mesenchymal stem cells facilitates endometrial regeneration and restores fertility. Acta Biomater. 92, 160–171. 10.1016/j.actbio.2019.05.012 31075515

[B150] Xin L. Lin X. Zhou F. Li C. Wang X. Yu H. (2020). A scaffold laden with mesenchymal stem cell-derived exosomes for promoting endometrium regeneration and fertility restoration through macrophage immunomodulation. Acta Biomater. 113, 252–266. 10.1016/j.actbio.2020.06.029 32574858

[B151] Xin L. Wei C. Tong X. Dai Y. Huang D. Chen J. (2022). *In situ* delivery of apoptotic bodies derived from mesenchymal stem cells via a hyaluronic acid hydrogel: A therapy for intrauterine adhesions. Bioact. Mater 12, 107–119. 10.1016/j.bioactmat.2021.10.025 35087967 PMC8777284

[B152] Xing H. Zhang Z. Mao Q. Wang C. Zhou Y. Zhou X. (2021). Injectable exosome-functionalized extracellular matrix hydrogel for metabolism balance and pyroptosis regulation in intervertebral disc degeneration. J. Nanobiotechnology 19 (1), 264. 10.1186/s12951-021-00991-5 34488795 PMC8419940

[B153] Xiong Y. Chen L. Liu P. Yu T. Lin C. Yan C. (2022). All-in-One: multifunctional hydrogel accelerates oxidative diabetic wound healing through timed-release of exosome and fibroblast growth factor. Small 18 (1), e2104229. 10.1002/smll.202104229 34791802

[B154] Xu H. L. Xu J. Shen B. X. Zhang S. S. Jin B. H. Zhu Q. Y. (2017a). Dual regulations of thermosensitive heparin-poloxamer hydrogel using epsilon-polylysine: bioadhesivity and controlled KGF release for enhancing wound healing of endometrial injury. ACS Appl. Mater Interfaces 9 (35), 29580–29594. 10.1021/acsami.7b10211 28809108

[B155] Xu H. L. Xu J. Zhang S. S. Zhu Q. Y. Jin B. H. ZhuGe D. L. (2017b). Temperature-sensitive heparin-modified poloxamer hydrogel with affinity to KGF facilitate the morphologic and functional recovery of the injured rat uterus. Drug Deliv. 24 (1), 867–881. 10.1080/10717544.2017.1333173 28574291 PMC8241134

[B156] Xu L. Chen Y. Zhang P. Tang J. Xue Y. Luo H. (2022). 3D printed heterogeneous hybrid hydrogel scaffolds for sequential tumor photothermal-chemotherapy and wound healing. Biomater. Sci. 10 (19), 5648–5661. 10.1039/d2bm00903j 35994007

[B157] Xu L. Ding L. Wang L. Cao Y. Zhu H. Lu J. (2017c). Umbilical cord-derived mesenchymal stem cells on scaffolds facilitate collagen degradation via upregulation of MMP-9 in rat uterine scars. Stem Cell Res. Ther. 8 (1), 84. 10.1186/s13287-017-0535-0 28420433 PMC5395893

[B158] Yan C. Chen J. Wang C. Yuan M. Kang Y. Wu Z. (2022). Milk exosomes-mediated miR-31-5p delivery accelerates diabetic wound healing through promoting angiogenesis. Drug Deliv. 29 (1), 214–228. 10.1080/10717544.2021.2023699 34985397 PMC8741248

[B159] Yang B. Chen Y. Shi J. (2019). Exosome biochemistry and advanced nanotechnology for next-generation theranostic platforms. Adv. Mater 31 (2), e1802896. 10.1002/adma.201802896 30126052

[B160] Yang H. Wu S. Feng R. Huang J. Liu L. Liu F. (2017). Vitamin C plus hydrogel facilitates bone marrow stromal cell-mediated endometrium regeneration in rats. Stem Cell Res. Ther. 8 (1), 267. 10.1186/s13287-017-0718-8 29157289 PMC5697119

[B161] Yang J. Chen Z. Pan D. Li H. Shen J. (2020). <p&gt;Umbilical cord-derived mesenchymal stem cell-derived exosomes combined pluronic F127 hydrogel promote chronic diabetic wound healing and complete skin regeneration</p&gt;. Int. J. Nanomedicine 15, 5911–5926. 10.2147/IJN.S249129 32848396 PMC7429232

[B162] Yang L. Ma N. Song D. Huang X. Zhou Q. Guo Y. (2022). The effect of estrogen in the prevention of adhesion reformation after hysteroscopic adhesiolysis: A prospective randomized control trial. J. Minim. Invasive Gynecol. 29 (7), 871–878. 10.1016/j.jmig.2022.04.004 35439645

[B163] Yao Q. Zheng Y. W. Lan Q. H. Wang L. F. Huang Z. W. Chen R. (2020). Aloe/poloxamer hydrogel as an injectable beta-estradiol delivery scaffold with multi-therapeutic effects to promote endometrial regeneration for intrauterine adhesion treatment. Eur. J. Pharm. Sci. 148, 105316. 10.1016/j.ejps.2020.105316 32201342

[B164] Yao Y. Chen R. Wang G. Zhang Y. Liu F. (2019). Exosomes derived from mesenchymal stem cells reverse EMT via TGF-β1/Smad pathway and promote repair of damaged endometrium. Stem Cell Res. Ther. 10 (1), 225. 10.1186/s13287-019-1332-8 31358049 PMC6664513

[B165] You Q. Lu M. Li Z. Zhou Y. Tu C. (2022). Cell sheet technology as an engineering-based approach to bone regeneration. Int. J. Nanomedicine 17, 6491–6511. 10.2147/ijn.S382115 36573205 PMC9789707

[B166] Yu K. Huang Z. Y. Xu X. L. Li J. Fu X. W. Deng S. L. (2022). Estrogen receptor function: impact on the human endometrium. Front. Endocrinol. (Lausanne) 13, 827724. 10.3389/fendo.2022.827724 35295981 PMC8920307

[B167] Yu M. Liu W. Li J. Lu J. Lu H. Jia W. (2020). Exosomes derived from atorvastatin-pretreated MSC accelerate diabetic wound repair by enhancing angiogenesis via AKT/eNOS pathway. Stem Cell Res. Ther. 11 (1), 350. 10.1186/s13287-020-01824-2 32787917 PMC7425015

[B168] Yuan L. Cao J. Hu M. Xu D. Li Y. Zhao S. (2022a). Bone marrow mesenchymal stem cells combined with estrogen synergistically promote endometrial regeneration and reverse EMT via Wnt/β-catenin signaling pathway. Reprod. Biol. Endocrinol. 20 (1), 121. 10.1186/s12958-022-00988-1 35971112 PMC9377128

[B169] Yuan M. Liu K. Jiang T. Li S. Chen J. Wu Z. (2022b). GelMA/PEGDA microneedles patch loaded with HUVECs-derived exosomes and Tazarotene promote diabetic wound healing. J. Nanobiotechnology 20 (1), 147. 10.1186/s12951-022-01354-4 35305648 PMC8934449

[B170] Zhang F. X. Liu P. Ding W. Meng Q. B. Su D. H. Zhang Q. C. (2021a). Injectable Mussel-Inspired highly adhesive hydrogel with exosomes for endogenous cell recruitment and cartilage defect regeneration. Biomaterials 278, 121169. 10.1016/j.biomaterials.2021.121169 34626937

[B171] Zhang L. Li Y. Guan C. Y. Tian S. Lv X. D. Li J. H. (2018). Therapeutic effect of human umbilical cord-derived mesenchymal stem cells on injured rat endometrium during its chronic phase. Stem Cell Res. Ther. 9 (1), 36. 10.1186/s13287-018-0777-5 29433563 PMC5810045

[B172] Zhang L. Wang M. Zhang Q. Zhao W. Yang B. Shang H. (2019a). Estrogen therapy before hysteroscopic adhesiolysis improves the fertility outcome in patients with intrauterine adhesions. Arch. Gynecol. Obstet. 300 (4), 933–939. 10.1007/s00404-019-05249-y 31350664

[B173] Zhang S. Chen L. Zhang G. Zhang B. (2020a). Umbilical cord-matrix stem cells induce the functional restoration of vascular endothelial cells and enhance skin wound healing in diabetic mice via the polarized macrophages. Stem Cell Res. Ther. 11 (1), 39. 10.1186/s13287-020-1561-x 31992364 PMC6986138

[B174] Zhang S. Li P. Yuan Z. Tan J. (2019b). Platelet-rich plasma improves therapeutic effects of menstrual blood-derived stromal cells in rat model of intrauterine adhesion. Stem Cell Res. Ther. 10 (1), 61. 10.1186/s13287-019-1155-7 30770774 PMC6377773

[B175] Zhang S. S. Xia W. T. Xu J. Xu H. L. Lu C. T. Zhao Y. Z. (2017). Three-dimensional structure micelles of heparin-poloxamer improve the therapeutic effect of 17&amp;beta;-estradiol on endometrial regeneration for intrauterine adhesions in a rat model. Int. J. Nanomedicine 12, 5643–5657. 10.2147/IJN.S137237 28848344 PMC5557621

[B176] Zhang S. S. Xu X. X. Xiang W. W. Zhang H. H. Lin H. L. Shen L. E. (2020b). Using 17β‐estradiol heparin‐poloxamer thermosensitive hydrogel to enhance the endometrial regeneration and functional recovery of intrauterine adhesions in a rat model. FASEB J. 34 (1), 446–457. 10.1096/fj.201901603RR 31914682

[B177] Zhang Y. Bi J. Huang J. Tang Y. Du S. Li P. (2020c). <p&gt;Exosome: A review of its classification, isolation techniques, storage, diagnostic and targeted therapy applications</p&gt;. Int. J. Nanomedicine 15, 6917–6934. 10.2147/IJN.S264498 33061359 PMC7519827

[B178] Zhang Y. Shi L. Lin X. Zhou F. Xin L. Xu W. (2021b). Unresponsive thin endometrium caused by asherman syndrome treated with umbilical cord mesenchymal stem cells on collagen scaffolds: A pilot study. Stem Cell Res. Ther. 12 (1), 420. 10.1186/s13287-021-02499-z 34294152 PMC8296628

[B179] Zhang Y. Xie Y. Hao Z. Zhou P. Wang P. Fang S. (2021c). Umbilical mesenchymal stem cell-derived exosome-encapsulated hydrogels accelerate bone repair by enhancing angiogenesis. ACS Appl. Mater Interfaces 13 (16), 18472–18487. 10.1021/acsami.0c22671 33856781

[B180] Zhou Q. Shi X. Saravelos S. Huang X. Zhao Y. Huang R. (2021). Auto-Cross-linked hyaluronic acid gel for prevention of intrauterine adhesions after hysteroscopic adhesiolysis: A randomized controlled trial. J. Minim. Invasive Gynecol. 28 (2), 307–313. 10.1016/j.jmig.2020.06.030 32681996

[B181] Zhou Y. Zhang X. L. Lu S. T. Zhang N. Y. Zhang H. J. Zhang J. (2022). Human adipose-derived mesenchymal stem cells-derived exosomes encapsulated in pluronic F127 hydrogel promote wound healing and regeneration. Stem Cell Res. Ther. 13 (1), 407. 10.1186/s13287-022-02980-3 35941707 PMC9358082

[B182] Zhu H. Pan Y. Jiang Y. Li J. Zhang Y. Zhang S. (2019). Activation of the Hippo/TAZ pathway is required for menstrual stem cells to suppress myofibroblast and inhibit transforming growth factor beta signaling in human endometrial stromal cells. Hum. Reprod. 34 (4), 635–645. 10.1093/humrep/dez001 30715393

[B183] Zhu Q. Tang S. Zhu Y. Chen D. Huang J. Lin J. (2022). Exosomes derived from CTF1-modified bone marrow stem cells promote endometrial regeneration and restore fertility. Front. Bioeng. Biotechnol. 10, 868734. 10.3389/fbioe.2022.868734 35497344 PMC9043110

[B184] Zhuang J. Hang R. Sun R. Ding Y. Yao X. Hang R. (2022). Multifunctional exosomes derived from bone marrow stem cells for fulfilled osseointegration. Front. Chem. 10, 984131. 10.3389/fchem.2022.984131 36072705 PMC9441814

[B185] Zou J. Yang W. Cui W. Li C. Ma C. Ji X. (2023). Therapeutic potential and mechanisms of mesenchymal stem cell-derived exosomes as bioactive materials in tendon-bone healing. J. Nanobiotechnology 21 (1), 14. 10.1186/s12951-023-01778-6 36642728 PMC9841717

